# Disparate oxidant gene expression of airway epithelium compared to alveolar macrophages in smokers

**DOI:** 10.1186/1465-9921-10-111

**Published:** 2009-11-17

**Authors:** Brendan J Carolan, Ben-Gary Harvey, Neil R Hackett, Timothy P O'Connor, Patricia A Cassano, Ronald G Crystal

**Affiliations:** 1 Department of Genetic Medicine, Weill Cornell Medical College, New York, New York, USA; 2 Division of Pulmonary and Critical Care Medicine, Weill Cornell Medical College, New York, New York, USA; 3 Division of Nutritional Sciences, Cornell University, Ithaca, New York, USA

## Abstract

**Background:**

The small airway epithelium and alveolar macrophages are exposed to oxidants in cigarette smoke leading to epithelial dysfunction and macrophage activation. In this context, we asked: what is the transcriptome of oxidant-related genes in small airway epithelium and alveolar macrophages, and does their response differ substantially to inhaled cigarette smoke?

**Methods:**

Using microarray analysis, with TaqMan RT-PCR confirmation, we assessed oxidant-related gene expression in small airway epithelium and alveolar macrophages from the same healthy nonsmoker and smoker individuals.

**Results:**

Of 155 genes surveyed, 87 (56%) were expressed in both cell populations in nonsmokers, with higher expression in alveolar macrophages (43%) compared to airway epithelium (24%). In smokers, there were 15 genes (10%) up-regulated and 7 genes (5%) down-regulated in airway epithelium, but only 3 (2%) up-regulated and 2 (1%) down-regulated in alveolar macrophages. Pathway analysis of airway epithelium showed oxidant pathways dominated, but in alveolar macrophages immune pathways dominated.

**Conclusion:**

Thus, the response of different cell-types with an identical genome exposed to the same stress of smoking is different; responses of alveolar macrophages are more subdued than those of airway epithelium. These findings are consistent with the observation that, while the small airway epithelium is vulnerable, alveolar macrophages are not "diseased" in response to smoking.

**Trial Registration:**

ClinicalTrials.gov ID: NCT00224185 and NCT00224198

## Introduction

Oxidants, free radicals with one or more unpaired electrons that are highly reactive, remove electrons from other molecules, changing their structure and function [1]. Cigarette smoking, with its estimated 10^14 ^free radicals per puff, creates a significant oxidant burden on the epithelial surface of the lung [2,3]. These oxidants are capable of modifying the structure and function of cellular and noncellular components, and in some cell populations, these modifications result in cell dysfunction and injury [1,4,5]. As in other organs, the potential of oxidants to damage pulmonary tissue is related to local antioxidant defense mechanisms, which transform free radicals into less reactive species, thereby limiting their toxic effects [6-9].

There are extensive data demonstrating that two cell populations on the respiratory epithelial surface, the small airway epithelium and alveolar macrophages, are involved in the pathogenesis of chronic obstructive pulmonary disease (COPD) associated with cigarette smoking [10-13]. The airway epithelium, endoderm-derived cells that form a continuous single cell barrier to the bronchial tree, responds to cigarette smoke exposure by up- and down-regulating a variety of oxidant-related genes, but eventually succumbs to the oxidant stress of smoking, becoming disordered in cell differentiation, repair and function [14-18]. In contrast, alveolar macrophages, mesoderm-derived phagocytic cells capable of releasing oxidants when activated, respond in a hierarchical fashion to incremental levels of oxidative stress, becoming activated, and play a role in mediating damage to other cells but do not become diseased *per se *[10,19,20]. In this context, we asked the question: with the knowledge that both the small airway epithelium and alveolar macrophages are exposed to the same oxidant stress of cigarette smoke and that the small airway epithelium becomes disordered and dysfunctional while alveolar macrophages become activated, are there differences in the program of oxidant-related gene expression in small airway epithelium and alveolar macrophages in response to smoking?

To address this question, we capitalized on the ability to obtain paired samples of small airway epithelium and alveolar macrophages from healthy nonsmokers and healthy smokers, thus circumventing the variability of genetic diversity and differences in smoking patterns among individuals. Using microarray gene expression analysis we compared oxidant-related gene expression in both cell populations for smokers and nonsmokers and the differences in response by smoking status. The data shows that at baseline in healthy nonsmokers, many oxidant-related genes are expressed at higher levels in alveolar macrophages than in small airway epithelium. However, in healthy smokers, in response to the stress of smoking, only a few oxidant-related genes responded in a similar fashion between the two cell types. There were far more smoking-induced changes in expression of oxidant-related genes in small airway epithelium than in alveolar macrophages This disparate response to cigarette smoking was also observed in analysis of functional pathways affected by smoking across all smoking responsive genes. Oxidation-related pathways predominated only in small airway epithelium and not in alveolar macrophages. Thus, in the same individuals, different cell-types with an identical genome and exposed to the same oxidant stress of cigarette smoking have very different responses. The changes in oxidant-related gene expression of alveolar macrophages were much less than those observed in small airway epithelium, suggesting that the small airway epithelium transcriptome is more responsive than alveolar macrophages to oxidative stress.

## Methods

### Study Population

Healthy nonsmokers and healthy smokers were recruited using local print media. The study was approved by the Weill Cornell Medical College Institutional Review Board, with written informed consent obtained from each individual before enrollment. Subjects were evaluated at the Weill Cornell NIH General Clinical Research Center and Department of Genetic Medicine Clinical Research Facility. Individuals were determined to be healthy on the basis of clinical history and physical examination, routine blood screening tests, urinalysis, chest X-ray, electrocardiogram and pulmonary function testing. Current smoking status was confirmed on history, venous carboxyhemoglobin levels, and urinalysis for nicotine levels and its derivative cotinine.

### Collection of Airway Epithelial Cells and Alveolar Macrophages

Small airway epithelial cells and alveolar macrophages were collected using flexible bronchoscopy [17,19]. Smokers were asked not to smoke for 12 hr prior to the procedure.

Small airway samples were collected from 10^th ^to 12^th ^order bronchi using methods previously described [17]. Briefly, a 2 mm diameter brush was wedged in the small airways of the right lower lobe and cells collected by gently brushing this area. These cells were subsequently collected in 5 ml of BEBM medium (GIBO, Grand Island, NY). An aliquot of this was used for cytology and differential cell count and the remainder was processed immediately for RNA extraction. Total cell number was determined by counting on a hemocytometer. Cell viability was estimated by Trypan Blue exclusion. Differential cell count was assessed on sedimented cells prepared by centrifugation (Cytospin 11; Shandon Instruments, Pittsburgh, PA) and stained with DiffQuik (Baxter Healthcare, Miami, FL).

Alveolar macrophages were collected by bronchoalveolar lavage (BAL), as previously described [19,21]. BAL fluid was centrifuged at 1,200 rpm for 5 min, 4°C. Cells were washed twice in RPMI 1640 containing 10% fetal bovine serum, 50 U/ml penicillin, 50 U/ml streptomycin and 2 mM glutamine (Invitrogen, Carlsbad, CA), suspended in 10 ml medium and an aliquot of 0.5 ml was used for total cell count, cell viability assessment and differential cell count. The remainder of the cells were seeded in six-well culture dishes (2 × 10^6 ^per 2 ml/well) and purified (≥97% alveolar macrophages) by adherence at 37°C, 2 hr in a 5% CO_2 _humidified incubator. Nonadherent cells were removed by washing with RPMI 1640 before RNA extraction.

### Selection of Oxidant-related Genes for Analysis

A list of oxidant-related genes was compiled from the literature and also by searching the Affymetrix associated gene annotations [6,7,22-24]. This list of oxidant-related genes was then categorized into major functional categories. Where multiple probe set identifications existed for a single oxidant-related gene, the probe set with the highest overall expression in small airway epithelium and alveolar macrophages was chosen to represent this gene. Data on all oxidant-related gene probe sets is available at the Gene Expression Omnibus (GEO) website.

### RNA Extraction and Microarray Processing and Analysis

Analyses were performed using Affymetrix (Santa Clara, CA) microarray HG-U133 Plus 2.0 (54,675 probe sets) and associated protocols. Total RNA was extracted from epithelial cells and alveolar macrophages using TRIzol (Invitrogen, Carlsbad, CA) followed by Rneasy (Qiagen, Valencia, CA) to remove residual DNA. This process yielded 2 to 4 μg RNA per 10^6 ^cells. An aliquot of each RNA sample was assessed using an Agilent Bioanalyzer (Agilent Technologies, Palo Alto, CA) to visualize and quantify the degree of RNA integrity. The concentration was determined using a NanoDrop ND-1000 spectrophotometer (NanoDrop Technologies, Wilmington, DE). Double stranded cDNA was synthesized from 3 μg of total RNA using the GeneChip One-Cycle cDNA Synthesis Kit, followed by cleanup with GeneChip Sample Cleanup Module, *in vitro *transcription (IVT) reaction using the GeneChip IVT Labeling Kit, and clean-up and quantification of the biotin-labeled cDNA yield by spectrophotometric analysis. All kits were from Affymetrix (Santa Clara, CA). Hybridizations to test chips and the microarrays were performed according to Affymetrix protocols, and microarrays were processed by the Affymetrix fluidics station and scanned with the Affymetrix GeneChip Scanner 3000 7G. Microarray quality was verified by the following criteria: (1) RNA Integrity Number (RIN) ≥7.0; (2) 3'/5' ratio for GAPDH ≤3; and (3) scaling factor ≤10.0[25,26]. Captured images were analyzed using the Microarray Suite version 5.0 (MAS 5.0) algorithm (Affymetrix). These data were normalized using GeneSpring version 6.2 software (Agilent Technologies) per array, by dividing raw data by the 50^th ^percentile of all measurements.

### TaqMan RT-PCR Confirmation of Microarray Gene Expression

cDNA was synthesized from 2 μg RNA in a 100 μl reaction volume, using the TaqMan Reverse Transcriptase Reaction Kit (Applied Biosystems, Foster City, CA), with random hexamers as primers. Then two dilutions of 1:10 and 1:100 were made from each sample and duplicate wells were run for each dilution. TaqMan PCR reactions were carried out using pre-made kits from Applied Biosystems and 2 μl of cDNA was used in each 25 μl reaction volume. The oxidant-related gene expression assays were optimized assays from Applied Biosystems. The endogenous control was 18S ribosomal RNA and relative expression levels were determined using the ΔΔCt method (Applied Biosystems) and the average value for the nonsmokers as the calibrator.

### Functional Pathway Assessment

To further assess the hypothesis that small airway epithelium is more oxidant-responsive than alveolar macrophages to the stress of smoking, analysis of pathways affected by smoking was done using Ingenuity Pathways Analysis http://www.ingenuity.com. All smoking responsive genes (fold-change >1.5 up- or down-regulated, p < 0.05 with Benjamini-Hochberg multiple test correction) were assessed using the pathway analysis program. Those canonical pathways chosen by the software analysis program on the basis of ratio (number of pathway genes in the smoking responsive data set compared to the total number of genes in the curated pathway) and significance (-log p value) were deemed those pathways most affected by cigarette smoking in small airway epithelium and alveolar macrophages.

### Statistical Analysis

A Chi-squared test was used to compare the distribution of gender and ethnicity between nonsmokers and smokers. The HG-U133 Plus 2.0 microarrays were analyzed using GeneSpring software. Average expression values for oxidant-related genes in small airway epithelial cell samples and alveolar macrophages were calculated using normalized expression levels for healthy nonsmokers and healthy smokers. Benjamini-Hochberg correction was applied to limit the false discovery rate. When assessing expression in alveolar macrophages *vs *small airway epithelium, comparisons for each oxidant-related gene were done first per individual and then all individual ratios were used to calculate the average fold-change in alveolar macrophages *vs *airway epithelium. A paired two-tailed t test was used to test the statistical significance of differences. When assessing the difference between smokers and nonsmokers, fold-change was calculated from the average expression values of smokers compared to the average expression value of nonsmokers and an unpaired two-tailed t test was used to test the statistical significance of differences. Fold-change was considered statistically significant if the magnitude of the fold-change was greater than 1.5 and p value < 0.05, respectively. Standard error was calculated for the fold change of smokers vs nonsmokers using the method of calculation of the standard error for a ratio.

### Web Deposition of Data

Data for the complete microarray study has been deposited in the Gene Expression Omnibus (GEO) site, http://www.ncbi.nlm.nih.gov/geo which is curated by the National Center for Bioinformatics (NCBI). Accession number is as follows: GSE13931.

## Results

### Study Population

Small airway samples and alveolar macrophages were collected from 19 healthy nonsmokers and 30 healthy smokers (Table 1). All individuals had no significant prior medical history and a normal physical examination. There were no differences between the groups with regard to age (p > 0.5), gender (p > 0.4) or self-reported ancestry (p > 0.2). All individuals were HIV negative, with blood and urine parameters within normal ranges. Smokers had an average smoking history of 27 pack-yr. Urine nicotine, urine cotinine and venous blood carboxyhemoglobin levels confirmed current smoking status of these individuals. Both populations had normal chest X-rays. Pulmonary function testing revealed normal spirometry, lung volumes and diffusing capacity for carbon monoxide in healthy nonsmokers and healthy smokers.

**Table 1:** Study Population for Paired Small Airway Epithelium and Alveolar Macrophage Samples^1^

Parameter	Healthy nonsmokers	Healthy smokers
**Demographics**		
n	19	30
Sex (male/female)	15/4	22/8
Age (yr)	42 ± 2	43 ± 1
Race (B/W/H/)^2^	11/6/2	18/10/2
Smoking history (pack-yr)	0	27 ± 3
Urine nicotine (ng/ml)	Negative	775 ± 176
Urine cotinine (ng/ml)	Negative	969 ± 136
Venous CO-Hb^3^	0.7 ± 0.2	2.3 ± 0.4
		
**Pulmonary function parameters^4^**		
FVC	107 ± 2	110 ± 2
FEV1	100 ± 6	110 ± 2
FEV1/FVC	80 ± 1	82 ± 1
TLC	97 ± 2	100 ± 2
DLCO	94 ± 2	100 ± 2
		
**Small airway epithelium**		
Total number recovered ×10^6^	5.1 ± 0.4	6.7 ± 0.4
% epithelial	99 ± 1	99 ± 1
% inflammatory	1 ± 1	0
Epithelial cell differential		
% ciliated	73 ± 2	76 ± 2
% secretory	9 ± 1	8 ± 1
% basal	13 ± 2	12 ± 1
% undifferentiated	5 ± 1	5 ± 1
		
**Alveolar macrophages**		
Total number recovered ×10^6^	14 ± 1	32 ± 12
% alveolar macrophages after purification	>97%	>98%

^1 ^Data are presented as mean ± standard error.

^2 ^B = Black W = White H = Hispanic.

^3 ^Venous carboxyhemoglobin, a secondary marker of current smoking; nonsmokers <1.5%.

^4 ^FVC = forced vital capacity; FEV1 = forced expiratory volume in first second; DLCO = diffusion capacity for carbon monoxide; all data presented as % predicted except for FEV1/FVC presented as % observed.

### Sampling of Airway Epithelium and Alveolar Macrophages

Airway epithelial cells were obtained from the small (10^th ^to 12^th ^order) airways. The number of epithelial cells recovered ranged from 2.5 to 7.8 × 10^6 ^for the nonsmokers and 3.5 to 13.9 × 10^6 ^for the healthy smokers (p < 0.05; Table 1). In all cases >98% of cells recovered were epithelial cells. The various categories of airway epithelial cells were as expected from the small airways; there were no differences in the subtypes of airway epithelial cells among the nonsmokers and smokers (p > 0.1, all comparisons). Alveolar macrophages were obtained from the same healthy nonsmokers and healthy smokers. Total alveolar macrophage counts obtained ranged from 11.9 to 17.8 × 10^6 ^for the nonsmokers and 8.3 to 32.4 × 10^6 ^for the healthy smokers; on average, more alveolar macrophages were recovered from healthy smokers than nonsmokers (p < 0.01). After purification, alveolar macrophages from both groups were ≥97% pure.

### Oxidant-related Gene Expression in Nonsmokers

**Figure 1:**
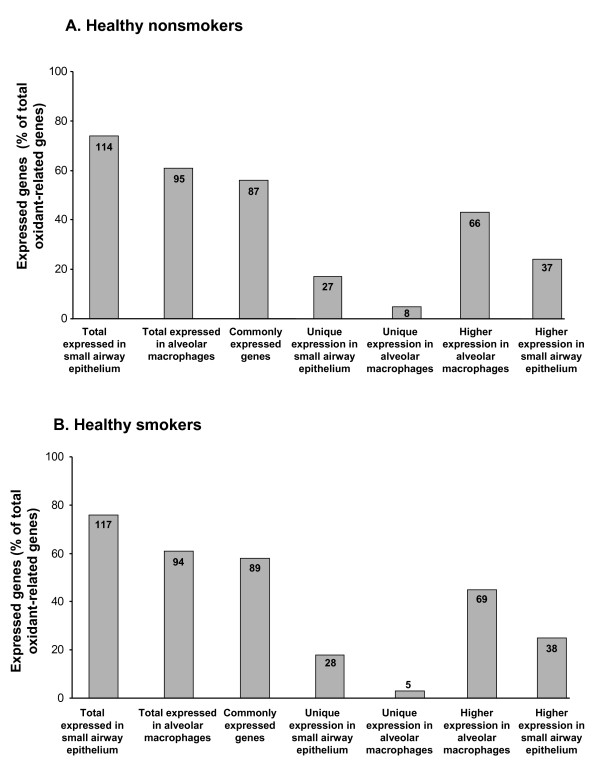
**Expression of oxidant-related genes in small airway epithelium and alveolar macrophages of healthy nonsmokers and healthy smokers**. **A**. Expression of oxidant-related genes in healthy nonsmokers. The % of all oxidant-related genes (total of 155 genes surveyed) that were expressed (defined as Affymetrix detection call of "present" in >50% of nonsmoker individuals, n = 19) is presented on the ordinate and the categories in which these genes are expressed are presented on the abscissa. Each bar represents the % of genes expressed and inside each bar is the corresponding number of oxidant-related genes expressed. **B**. Expression of oxidant-related genes in healthy smokers. The % of all oxidant-related genes that were expressed in healthy smokers (n = 30) is presented on the ordinate and the categories in which these genes are expressed is presented on the abscissa. Each bar represents the % of genes expressed and inside each bar is the corresponding number of oxidant-related genes expressed.

To describe the expression patterns of oxidant-related genes in small airway epithelium and alveolar macrophages, a list of 155 oxidant-related genes with probes on the Affymetrix HG-U133 Plus 2.0 microarray was compiled from the literature and categorized by function or pathway (Additional File 1). These genes included: glutathione metabolism (35 genes), redox balance (3), catalase/superoxide dismutase (4), oxidant scavengers (24), pentose phosphate cycle (14), xenobiotic metabolism (58), selenium-related (7), bilirubin-related (4), ascorbic acid-related (2) and production of free radicals (4). Using an expression criterion of having an Affymetrix Detection Call of "Present" in ≥50% of individuals, genes from most of these categories were expressed in both small airway epithelium and alveolar macrophages of nonsmokers. Of the total 155 oxidant-related genes surveyed in the nonsmokers, 87 genes (56%) were expressed in both cell types (Additional File 1; Figure 1). There were 27 (17%) oxidant-related genes expressed only in small airway epithelium that were not expressed in alveolar macrophages of nonsmokers; 21 (78%) of these genes were in the xenobiotic metabolism category and 4 (15%) genes were in the glutathione metabolism category. However, there were only 8 (5%) oxidant-related genes expressed in alveolar macrophages that were not expressed in small airway epithelium; 5 of these genes were in the xenobiotic metabolism category. Among those genes uniquely expressed in small airway epithelium were xenobiotic genes CYP4F3, CYP4Z1, CYP7B1 and CYP39A1 that have not previously been described as expressed in human airway epithelium.

### Oxidant-related Gene Expression in Smokers

Of the 155 oxidant-related genes surveyed, there were 89 (57%) genes expressed in both the small airway epithelium and alveolar macrophages of healthy smokers. Similar to healthy nonsmokers, there were 28 (18%) oxidant-related genes expressed only in the small airway epithelium of smokers and not expressed in alveolar macrophages from these same individuals. There were 5 (3%) oxidant-related genes whose expression was unique to the alveolar macrophages of smokers (Figure 1, Additional File 1).

### Differential Expression of Oxidant-related Genes in the Small Airway Epithelium and Alveolar Macrophages

While the majority of oxidant-related genes were expressed in both alveolar macrophages and small airway epithelium from healthy nonsmokers and healthy smokers, there were marked differences in expression levels of these genes between the two cell populations from these same individuals.

In the nonsmokers there were 122 (78%) genes that were expressed in either alveolar macrophages or small airway epithelium, and 103 genes (67%) had differential expression (fold change >1.5, p < 0.05) between the two cell populations. There were 37 (24%) genes with significantly decreased expression in alveolar macrophages compared to small airway epithelium, 24 of these were not expressed at all in alveolar macrophages. However, 66 (43%) genes had higher expression levels in alveolar macrophages compared to small airway epithelium in these same nonsmokers and only 7 of these were explained by absence of expression in small airway epithelium (Figure 1, Additional File 2, Additional File 3).

In healthy smokers, of the 123 genes that were expressed in either alveolar macrophages or small airway epithelium, there were 107 (69%) oxidant-related genes with differential expression (fold change >1.5, p < 0.05) between the two cell populations. There were 38 genes (25%) with higher expression levels in airway epithelium than in alveolar macrophages; 26 of these were explained by the absence of expression in alveolar macrophages. Similar to the nonsmokers, in the healthy smokers there were 69 (45%) genes with higher expression in alveolar macrophages compared to small airway epithelium. Only 6 of these genes lacked expression in small airway epithelium (Figure 1, Additional File 4 and Additional File 5).

**Figure 2:**
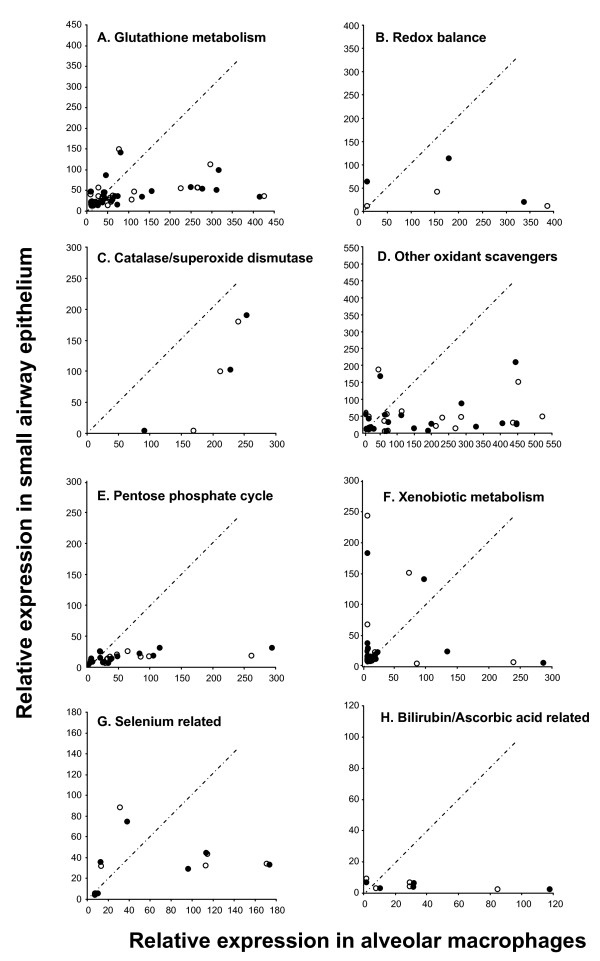
**Relative expression of oxidant-related genes in small airway epithelium compared to alveolar macrophages from the same healthy nonsmokers and healthy smokers**. Presented only are those oxidant-related genes expressed (Affymetrix detection call of present in >50% of small airway epithelium or alveolar macrophages) in healthy nonsmokers or healthy smokers. Average relative expression of oxidant-related genes in small airway epithelium is presented on the ordinate and average relative expression of oxidant-related genes in alveolar macrophages is presented on the abscissa. Each point represents one gene. white circle = average expression in healthy nonsmokers; black circle = average relative expression in healthy smokers. The different categories of oxidant-related genes surveyed are presented in panels **A-H**. **A**. Glutathione metabolism; **B**. Redox balance; **C**. Catalase/superoxide dismutases; **D**. Other oxidant scavengers; **E**. Pentose pathway cycle; **F**. Xenobiotic metabolism; **G**. Selenium-related; and **H**. Bilirubin/ascorbic acid related.

Overall there was higher expression in alveolar macrophages than in small airway epithelium for both nonsmokers and healthy smokers across all categories of oxidant-related genes. This was particularly striking in the categories of glutathione metabolism, redox balance and other oxidant scavengers (Figure 2).

### Smoking Responsive Oxidant-related Genes

**Figure 3:**
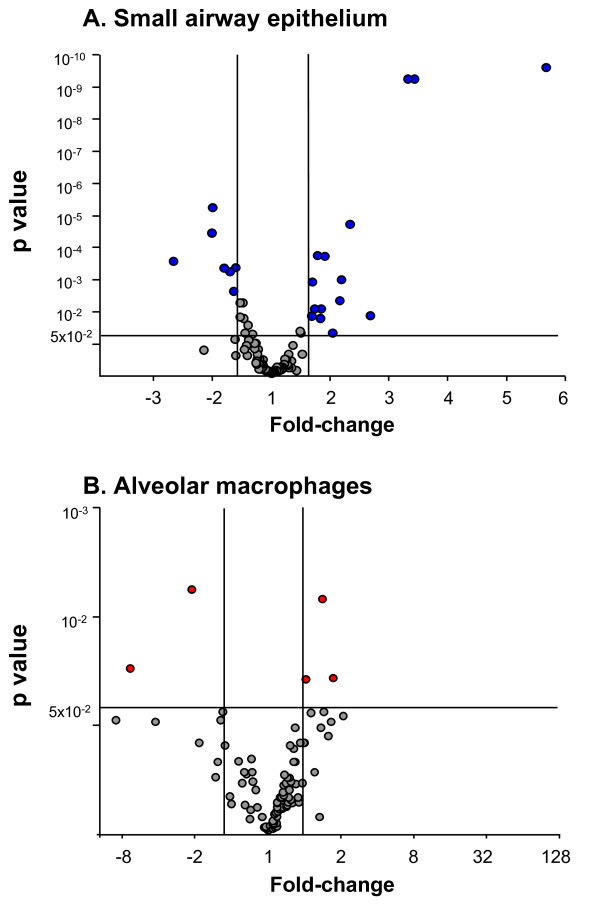
**Smoking responsiveness of oxidant-related genes expressed in the small airway epithelium and alveolar macrophages of healthy nonsmokers (n = 19) and healthy smokers (n = 30)**. **A**. Smoking responsiveness of expressed oxidant-related genes in small airway epithelium. **B**. Smoking responsiveness of expressed oxidant-related genes in alveolar macrophages. For both **A **and **B**, the ordinate shows p value and the abscissa shows fold-change. Each data point represents 1 oxidant-related gene. Grey = oxidant-related genes that are not significantly changed (p > 0.05 and/or fold-change up or down <1.5) in small airway epithelium or alveolar macrophages of normal smokers compared to normal nonsmokers. Blue = oxidant-related genes that are smoking responsive or significantly changed (p < 0.05, fold change up or down ≥1.5) in the small airway epithelium; red = oxidant-related genes that are smoking responsive or significantly changed in alveolar macrophages of healthy smokers compared to healthy nonsmokers.

**Figure 4:**
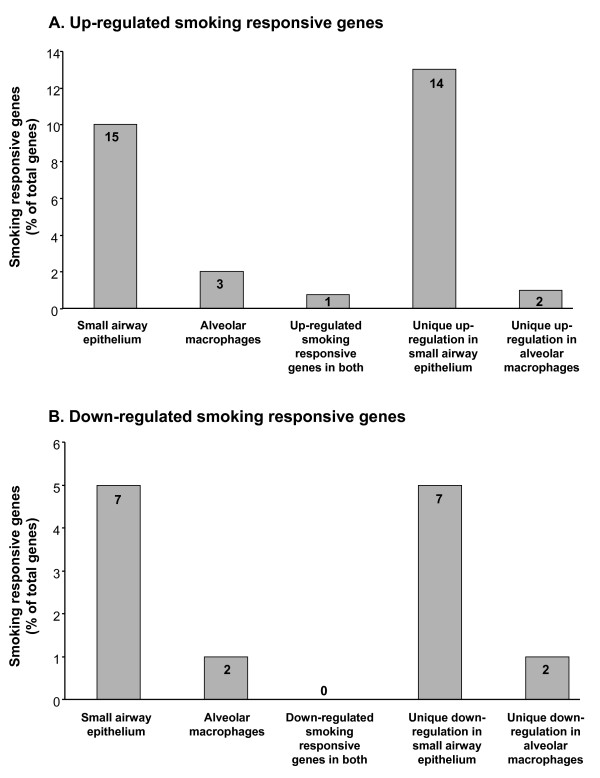
**Percentage of the total of 155 oxidant-related genes surveyed that are smoking responsive in small airway epithelium and alveolar macrophages**. **A**. Oxidant-related genes whose expression is up-regulated in healthy smokers compared to healthy nonsmokers. **B**. Oxidant-related genes whose expression is down-regulated in healthy smokers compared to healthy nonsmokers. For both **A **and **B**, the percentage of oxidant-related genes up or down-regulated are presented on the ordinate and the different categories are presented on the abscissa; within each bar is the number of genes modified.

There was a similar number of oxidant-related genes expressed in small airway epithelium and alveolar macrophages of healthy nonsmokers and healthy smokers, but there were marked differences in the pattern of genes that were up- or down-regulated by smoking. Using a criterion for smoking responsiveness of a statistically significant (p < 0.05) fold-change (increase or decrease of ≥1.5) in healthy smokers compared to nonsmokers, the expression of 22 oxidant-related genes was up- or down-regulated in the small airway epithelium and 5 genes were up- or down-regulated in alveolar macrophages (Figure 3, Tables 2, 3). In that context, the expression of 15 oxidant-related genes was up-regulated in the small airway epithelium, and only 1 of these genes was also up-regulated in alveolar macrophages. In contrast, only 3 genes were up-regulated by smoking in alveolar macrophages, with 2 of them unique to alveolar macrophages. Seven genes were down-regulated in the small airway epithelium of smokers; all of these genes were uniquely down-regulated in small airway epithelium, and an additional 2 genes were uniquely down-regulated in alveolar macrophages from the same individuals (Figure 4).

**Table 2:** Smoking Responsive Oxidant-related Gene Expression in the Small Airway Epithelium^1^

Category	Probe set ID	Gene symbol	Gene title	Nonsmoker % present^2^	Healthy smoker % present	Smokers/nonsmokers (fold-change)	p value
Glutathione metabolism	202275_at	G6PD	glucose-6-phosphate dehydrogenase	42	77	1.78	0.015
	**202923_s_at**	**GCLC**	**glutamate-cysteine ligase, catalytic subunit**	**100**	**100**	**1.50**	**0.005**
	202831_at	GPX2	glutathione peroxidase 2	100	100	5.00	< 0.001
	205770_at	GSR	glutathione reductase	100	100	1.60	0.013
Redox balance	210505_at	ADH7	alcohol dehydrogenase 7, mu or sigma polypeptide	100	100	5.37	< 0.001
	201272_at	AKR1B1	aldo-keto reductase family 1, member B1	100	100	1.72	< 0.001
	209160_at	AKR1C3	aldo-keto reductase family 1, member C3	100	100	2.53	< 0.001
Catalase/SOD	No smoking responsive genes in this category in small airway epithelium						
Other oxidants scavengers	202018_s_at	LTF	lactotransferrin	100	87	-3.16	< 0.001
	212859_x_at	MT1E	metallothionein 1E	100	100	-1.75	< 0.001
	213629_x_at	MT1F	metallothionein 1F	100	100	-2.00	< 0.001
	208581_x_at	MT1X	metallothionein 1X	100	100	-1.56	0.002
	212185_x_at	MT2A	metallothionein 2A	100	100	-1.64	0.001
	201266_at	TXNRD1	thioredoxin reductase 1	100	100	1.87	< 0.001
Pentose phosphate cycle	201118_at	PGD	phosphogluconate dehydrogenase	100	100	1.66	0.008
	**213093_at**	**PRKCA**	**protein kinase C, alpha**	**100**	**100**	**-1.52**	** < 0.001**
	201463_s_at	TALDO1	transaldolase 1	100	100	1.61	0.001
	208699_x_at	TKT	transketolase	100	100	1.79	0.008
Xenobiotic metabolism	202437_s_at	CYP1B1	cytochrome P450, family 1, subfamily B, polypeptide 1	32	97	25.41	< 0.001
	206515_at	CYP4F3	cytochrome P450, family 4, subfamily F, polypeptide 3	95	93	2.27	0.001
	206153_at	CYP4F11	cytochrome P450, family 4, subfamily F, polypeptide 11	79	90	3.21	0.013
	227702_at	CYP4X1	cytochrome P450, family 4, subfamily X, polypeptide 1	100	100	-2.02	< 0.001
	206424_at	CYP26A1	cytochrome P450, family 26, subfamily A, polypeptide 1	37	63	2.05	0.045
Bilirubin related	No smoking responsive genes in this category in small airway epithelium						
Ascorbic acid related	No smoking responsive genes in this category in small airway epithelium						
Production of free radicals	No smoking responsive genes in this category in small airway epithelium						

^1 ^Smoking responsive genes defined as fold-change smokers compared to nonsmokers >1.5 and p value < 0.05 in genes expressed in >50% of either nonsmokers or smokers. Bold type represents differentially expressed genes in both small airway epithelium and alveolar macrophages (see Table 3).

^2 ^% present refers to Affymetrix Detection Call of Present as a % of total numbers of healthy nonsmokers (n = 19) and healthy smokers (n = 30).

**Table 3:** Smoking Responsive Oxidant-related Gene Expression in Alveolar Macrophages^1^

Category	Probe set ID	Gene symbol	Gene title	Nonsmokers % present^2^	Healthy smokers % present	Smokers/non-smokers (fold-change)	p value
Glutathione metabolism	**202923_s_at**	**GCLC**	**glutamate-cysteine ligase, catalytic subunit**	**100**	**100**	**1.67**	**0.007**
Redox balance	No smoking responsive genes in this category in the alveolar macrophages						
Catalase/SOD	216841_s_at	SOD2	superoxide dismutase 2	100	100	-2.06	0.006
Oxidant scavengers	No smoking responsive genes in this category in the alveolar macrophages						
Pentose phosphate cycle	**213093_at**	**PRKCA**	**protein kinase C, alpha**	**95**	**100**	**1.86**	**0.037**
Xenobiotic metabolism	205939_at	CYP3A7	cytochrome P450, family 3, subfamily A, polypeptide 7	58	10	-3.69	0.030
Bilirubin related	209236_at	SLC23A2solute carrier family 23, member 2		89	100	1.50	0.038
Ascorbic acid related	No smoking responsive genes in this category in the alveolar macrophages						
Production of free radicals	No smoking responsive genes in this category in the alveolar macrophages						

^1 ^Smoking responsive genes defined as fold-change smokers compared to nonsmokers >1.5 and p value < 0.05 in genes expressed in >50% of either nonsmokers or smokers. Bold type represents differentially expressed genes in both small airway epithelium and alveolar macrophages (see Table 2).

^2 ^% present refers to Affymetrix Detection Call of Present as a % of total numbers of healthy nonsmokers (n = 19) and healthy smokers (n = 30).

**Figure 5:**
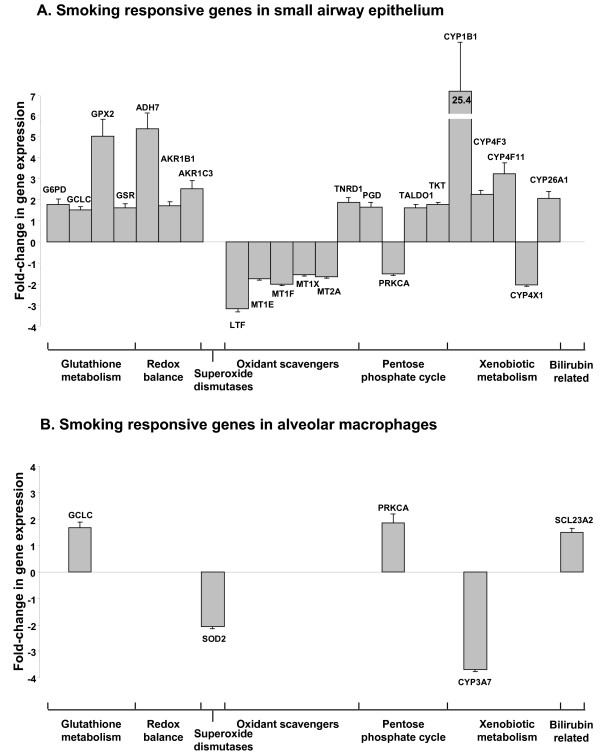
**Comparison of the fold-changes of smoking responsive oxidant-related genes in the small airway epithelium and alveolar macrophages in the different oxidant-related gene categories**. **A**. Fold-change of smoking responsive oxidant-related genes in small airway epithelium. **B**. Fold-change of smoking responsive oxidant-related genes in alveolar macrophages. For both **A **and **B**, the fold-change is presented on the ordinate and the oxidant-related genes in their categories on the abscissa. Each bar represents the fold-change (average expression in healthy smokers compared to average expression in healthy nonsmokers) of an oxidant related gene in the corresponding category and error bars represent the standard error for the ratio.

The differences between smokers and nonsmokers in the response of the small airway epithelium and alveolar macrophages were apparent when comparing the level of statistical significance (p value) and fold-change (up- or down-regulated) with far more dramatic changes apparent in the small airway epithelium. Whereas smoking-induced down-regulation was observed in both small airway epithelium and alveolar macrophages, this direction of response was far more common in the small airway epithelium, with the major down-regulation of expression in the category of oxidant scavengers (Figure 5, Table 2).

In the category of glutathione metabolism, 4 genes were up-regulated in the small airway epithelium (p < 0.02), but only one of these genes was also changed in alveolar macrophages (glutamate cysteine ligase, catalytic subunit, p < 0.01). Three genes were up-regulated in the redox balance category in small airway epithelium (p < 0.01), while there were no genes from this category changed significantly in alveolar macrophages. There were no changes in catalase/superoxide dismutase gene expression in small airway epithelium, but superoxide dismutase 2 was down-regulated in alveolar macrophages of smokers (p < 0.01). Interestingly, with regard to other oxidant scavengers, 5 genes were down-regulated in small airway epithelium and 1 gene was up-regulated while there was no apparent effect of smoking in this category in alveolar macrophages. Four genes from the pentose phosphate cycle were either up-or down-regulated in healthy smokers compared to nonsmokers in small airway epithelium (p < 0.01), but only 1 gene from this pathway changed significantly in alveolar macrophages (protein kinase C alpha, p < 0.04).

The expression of 5 genes involved in xenobiotic metabolism was significantly different in healthy smokers compared to healthy nonsmokers in small airway epithelium (p < 0.05). Four genes were upregulated in the small airway epithelium of smokers, including cytochrome P450, 1B1 (p < 0.01). Three other xenobiotic metabolism genes were also uniquely up-regulated in small airway epithelium including cytochrome P450 4F3, 4F11 and 26A1 (p < 0.05). There was 1 gene involved in xenobiotic metabolism that was uniquely down-regulated in small airway epithelium (cytochrome P450, 4X1, p < 0.001) and 1 gene that was uniquely down-regulated in alveolar macrophages, (cytochrome P450, 3A7, p < 0.04). In the category of bilirubin related genes, solute carrier 23A2 was uniquely up-regulated in alveolar macrophages (p < 0.04).

The gene expression pattern of 2 oxidant-related genes was altered in both the small airway epithelium and alveolar macrophages; glutamate cysteine ligase (p < 0.01 for both cell types) and protein kinase C alpha (p < 0.01 for both cell types, Figure 5). Interestingly, protein kinase C alpha, a member of the pentose phosphate cycle, was down-regulated in small airway epithelium of healthy smokers (1.5-fold decrease, p < 0.01) but up-regulated in alveolar macrophages of healthy smokers (1.9-fold increase, p < 0.04; Figure 5).

### TaqMan RT-PCR Confirmation of Microarray Gene Expression

To validate the results obtained from the microarray, TaqMan RT-PCR was carried out to assess several genes using RNA samples from healthy nonsmokers (n = 6) and healthy smokers (n = 6). In the small airway epithelium, TaqMan RT-PCR confirmed the up-regulation of alcohol dehydrogenase 7 (6.2 fold increase, p < 0.02), glutathione peroxidase 2 (3.9 fold increase, p < 0.01), cytochrome P450 1B1 (36.7 fold increase, p < 0.03) and down-regulation of metallothionein 1F (2.3 fold decrease, p < 0.05). In alveolar macrophages, TaqMan RT-PCR confirmed the down-regulation of superoxide dismutase 2 (3.6 fold decrease, p < 0.03) and the up-regulation of glutamate cysteine ligase catalytic subunit (3.4 fold increase, p < 0.01) and protein kinase C alpha (4.2 fold increase, p < 0.01, Table 4).

**Table 4:** TaqMan RT-PCR Confirmation of Smoking Responsive Genes in Small Airway Epithelium and Alveolar Macrophages

**Small airway epithelium**			**Alveolar macrophages**		
	
**Gene**	**Fold-change^1^**	**p value^2^**	**Gene**	**Fold-change**	**p value**
	
Alcohol dehydrogenase 7	6.2	< 0.02	Superoxide dismutase 2	-3.6	< 0.03
Glutathione peroxidase 2	3.9	< 0.01	Glutamate cysteine ligase C	3.8	< 0.01
Metallothionein 1F	-2.3	< 0.05	Protein kinase C alpha	4.2	< 0.01
Cytochrome P450 1B1	36.7	< 0.03			

^1 ^Fold change is calculated as the average expression level in healthy smokers (n = 6) compared to the average expression level in healthy nonsmokers (n = 6).

^2 ^p value calculated using an unpaired t test assuming unequal variance.

### Functional Pathway Assessment

To further assess the response of small airway epithelium and alveolar macrophages from the same individuals to cigarette smoke, all smoking responsive genes were examined using functional analysis software. In total, there were 297 smoking responsive genes in small airway epithelium and 116 smoking responsive genes in alveolar macrophages. Overall, the main pathways affected by smoking were related to xenobiotic metabolism and oxidant response in small airway epithelium while oxidation pathways did not feature as being enriched in alveolar macrophages (Table 5). The main canonical pathways affected in small airway epithelium were metabolism of xenobiotics by cytochrome p450 [ratio (number of pathway genes in the smoking responsive data set compared to the total number of genes in the pathway) 0.047, p < 0.001], xenobiotic metabolism signaling (ratio 0.048, p < 0.001), arachidonic acid metabolism (ratio 0.038, p < 0.001), pentose and glucuronate interconversions (ratio 0.047, p < 0.001) and glutathione metabolism (ratio 0.048, p < 0.001). In contrast, the main canonical pathways affected in alveolar macrophages were IL-10 signaling (ratio 0.07, p < 0.001), peroxisome proliferator activator receptor alpha (PPARa)/retinoid × receptor alpha (RXRa, ratio 0.038, p < 0.001), liver × receptor/RXR activation (ratio 0.059, p < 0.001), acute phase signaling (ratio 0.04, p < 0.001) and hepatic cholestasis (ratio 0.036, p < 0.001).

**Table 5:** Pathway Analysis of Smoking Responsive Genes in Small Airway Epithelium and Alveolar Macrophages^1^

**Small airway epithelium**			**Alveolar macrophages**		
	
**Functional pathway**	**Ratio^2^**	**p value**	**Functional pathway**	**Ratio**	**p value**
	
Xenobiotic metabolism	0.05	< 0.001	IL-10 signaling	0.07	< 0.001
Xenobiotic signaling	0.05	< 0.001	Peroxisome proliferator activator pathway/retinoic acid × receptor activation	0.04	< 0.001
Arachidonic acid metabolism	0.04	< 0.001	Liver × receptor activation pathway	0.06	< 0.001
Pentose and glucuronate interconversions	0.03	< 0.001	Acute phase signaling pathway	0.04	< 0.001
Glutathione metabolism	0.05	< 0.001	Hepatic cholestasis	0.04	< 0.001

^1 ^Functional pathway analysis was carried out using Ingenuity Pathway Analysis http://www.ingenuity.com on all smoking responsive (fold-change healthy smokers compared to healthy nonsmokers >1.5, p < 0.05 following Benjamini-Hochberg multiple test correction) genes in small airway epithelium and alveolar macrophages from the same healthy nonsmokers (n = 19) and healthy smokers (n = 30). Canonical pathways were selected on the basis of significance and ratio

^2 ^Ratio refers to the number of pathway genes in the smoking responsive dataset compared to the total number of genes in the curated pathway.

## Discussion

Cigarette smoking delivers a large oxidant burden to the epithelial surface of the lung, with consequent changes in the function of the epithelium and alveolar macrophages [2,3]. There are extensive data implicating airway epithelial cells and alveolar macrophages in the development of COPD, but their roles are very different [2,3,11,12]. Given that small airway epithelium becomes disorganized and dysfunctional in response to cigarette smoking [12,14,17], while alveolar macrophages become activated [10,19,20], we asked the question; are there differences in the gene expression patterns of oxidant-related genes in the small airway epithelium and alveolar macrophages of the same individuals and how do these different cell populations respond to the oxidant stress of smoking? Using microarray assessment the data demonstrates that the majority of oxidant-related genes are expressed in both cell types from the same individuals, but overall, the expression level of oxidant-related genes is higher in alveolar macrophages than small airway epithelium. Interestingly, however, airway epithelial cells show a much greater response to smoke exposure than alveolar macrophage cells. While cigarette smoking is associated with many significant differences in the expression of oxidant-related genes in small airway epithelial cells there are fewer smoking-related differences in oxidant-related gene expression in alveolar macrophages. These observations are consistent with the concept that the small airway epithelium is more responsive to the oxidant stimulus of cigarette smoke than alveolar macrophages, and consistent with the clinical observation that the small airway epithelium is associated with the earliest morphologic changes associated with smoking, whereas smoking does not cause "disease" of alveolar macrophages.

### Expression of Oxidant-related Genes

There are more oxidant-related genes expressed in the small airway epithelium than in alveolar macrophages of nonsmokers and smokers. Most of the uniquely expressed genes in the small airway epithelium relate to glutathione and xenobiotic metabolism, underlining the greater antioxidant and detoxifying role of the small airway epithelium. Within the category of xenobiotic metabolism there are a number of genes expressed that have not previously been described in human lung epithelium including CYP4F3, which is up-regulated by smoking in small airway epithelium. Interestingly, this cytochrome enzyme plays a role in the inactivation of leukotriene B4, a potent chemotaxin in the lung [27]. Based on the knowledge that xenobiotic metabolism represents an activating step in a number of pro-inflammatory and pro-carcinogen components of cigarette smoke, the finding of increased expression of cytochrome P450 enzymes in small airway epithelium compared to alveolar macrophages from the same individuals is consistent with the concept that the earliest site of disease in COPD is in the small airways [12,14].

Within an individual, the majority of the oxidant-related genes that are expressed in both cell populations are expressed at much higher levels in alveolar macrophages compared to small airway epithelium. Macrophages are responsive to low levels of oxidants, using them in signal transduction to activate transcription [28]. Given that alveolar macrophages use reactive oxygen species in the respiratory burst during phagocytosis [29], it may be important for cytoprotection to express high baseline levels of antioxidants in situations where endogenous oxidants pose a threat, thus rendering them less responsive to exogenous oxidant stress. However, when exposed to high concentrations of exogenous oxidants the oxidant/antioxidant imbalance can lead to stimulation of cell surface receptors which activate the inflammatory response in macrophages through activation of nuclear factor kappa B, mitogen activated protein kinase and activator protein 1 pathways further damaging the lung [28].

### Smoking Responsive Oxidant-related Genes

When examining the effects of smoking on oxidant-related gene expression there are far more differences in oxidant-related gene expression in small airway epithelium of healthy smokers compared to healthy nonsmokers than in alveolar macrophages from the same individuals. This suggests that the small airway epithelium may be more responsive to the oxidant burden of cigarette smoke than alveolar macrophages. Alveolar macrophages exhibit fewer changes in oxidant-related gene expression than airway epithelium; while this may be an artifact of the small sample size of individuals, it may also be because they are constantly being replaced in lung tissue and their cumulative exposure to oxidant stress may be less. However, the lifespan of an alveolar macrophage is a matter of debate, but these cells may reside in the lung for a relatively long period of time (months) and therefore have a similar turnover to small airway epithelium [29-31]. Another consideration is the physical location of alveolar macrophages, which reside primarily on the alveolar surface; thus, they are exposed to a more diluted form of cigarette smoke, being further away from the initial inhalation than airway epithelium. However, this is not the case when examining global changes in gene expression in alveolar macrophages where there are many changes across various categories of genes in healthy cigarette smokers [17,19]. An alternative explanation is that alveolar macrophages are relatively less responsive than small airway epithelium to exogenous oxidants. Consistent with this concept, primary human and murine alveolar macrophages are much less responsive to the pro-oxidant and pro-inflammatory effects of diesel exhaust particles than airway epithelial cell lines and normal human bronchial epithelial cells [32]. While epithelial cells more rapidly transitioned from a cytoprotective to cytotoxic response, alveolar macrophages responded in a hierarchal fashion, able to withstand higher concentrations of exogenous oxidants. This inherent resistance to oxidative stress in alveolar macrophages may be related to their ability to convert N-acetylcysteine to cytoprotective glutathione which epithelial cells cannot do [32]. In other studies, the *in vitro *response of rat alveolar macrophages and type II epithelial cells to paraquat exposure (a herbicide that injures lung cells by oxidant-related mechanisms including DNA strand breaks) showed significantly higher DNA strand breaks in type II epithelial cells compared to alveolar macrophages, suggesting that alveolar macrophages may have better intrinsic antioxidant mechanisms and/or have more efficient repair mechanisms following oxidant injury than epithelial cells [33]. These studies, together with the present study of *in vivo *cigarette smoking in humans, suggest that the available transcriptome of alveolar macrophages is relatively less sensitive than that of the small airway epithelium to the oxidant stress of cigarette smoke.

### Categories of Smoking-responsive Oxidant-related Genes

A similar number of the surveyed oxidant-related genes are expressed in both small airway epithelium and alveolar macrophages However, only the small airway epithelium responds to cigarette smoke by altering the gene expression profile of key pathways including glutathione metabolism, pentose phosphate cycle, redox balance and oxidant scavengers.

Glutathione is a ubiquitous tripeptide with a sulfhydryl group that enables it to protect cells from oxidant damage, and therefore serves as a major antioxidant in the lungs [9]. Glutathione homeostasis is regulated by glutathione cysteine ligase (GCLC) and the glutathione redox system. Consistent with previous studies, we observed increased gene expression of many enzymes involved in glutathione metabolism, including GCLC, in airway epithelium of healthy smokers [15,18]. Related to this is the pentose phosphate cycle, which produces reducing equivalents in the form of NADPH necessary for the generation of reduced glutathione [34]. Similar to what we observed, glucose-6 phosphate dehydrogenase and phosphogluconate dehydrogenase are also induced in rabbit lung following oxidant injury, indicating that these enzymes play a role in the regenerative response following acute oxidant injury [35]. While there are no studies currently linking members of the pentose phosphate cycle to the pathogenesis of COPD, protein kinase C alpha subunit (PRKCA) is down-regulated in small airway epithelium of smokers but up-regulated in alveolar macrophages from the same individuals. PRKC increases activation of nuclear factor erythroid 2-related factor 2 (NRF2), an oxidant responding transcription factor known to induce phase 2 detoxifying and antioxidant gene expression to protect cells from oxidative stress [36]. Studies have shown decreased NRF2 protein in the lungs of smokers who develop COPD [37], and in alveolar macrophages from smokers with emphysema [38].

In the category of oxidant scavengers, the expression of a number of metallothioneins is down-regulated in small airway epithelium in response to smoking. Overexpression of metallothioneins decreases sensitivity of pulmonary endothelial cells to oxidant injury [39], and while increased expression occurs in rat bronchial epithelial cells in response to acute cigarette smoke exposure, the effect is attenuated by chronic smoke exposure [39,40]. In our study, and consistent with other studies, decreased gene expression of metallothioneins in chronic healthy smokers may contribute to making the epithelium more vulnerable to oxidative damage than alveolar macrophages [16].

Superoxide dismutases are scavengers of free radicals within the cell. In alveolar macrophages superoxide dismutase 2 is down-regulated by smoking. There is conflicting evidence regarding the effects of smoking on SOD2, with some studies demonstrating up-regulation in smokers and others demonstrating down-regulation of the SOD2 protein, including down-regulation of expression in alveolar macrophages of healthy smokers compared to nonsmokers [41-43].

### Functional Pathway Assessment

Functional pathway analysis of all smoking responsive genes in small airway epithelium and alveolar macrophages revealed that the dominant canonical pathways affected by smoking in small airway epithelium were related to detoxifying and oxidant responses. However, IL-10, peroxisome proliferator activator receptor (PPAR) and liver × receptor pathways (LXR), are the top three pathways enriched in alveolar macrophages of healthy smokers. IL-10 plays a role in immune tolerance and pathogen clearance [44], PPAR down-regulates the synthesis of immunomodulatory cytokines and PPAR agonists may have a protective role in oxidative stress [45], while LXR inhibits macrophage responses to bacterial pathogens and antagonizes a number of pro-inflammatory cytokines [46]. These functional pathway data are further confirmation of the hypothesis that the small airway epithelium is the main site of oxidant response in the lung while the oxidant response of alveolar macrophages is relatively less given the same *in vivo *stress of cigarette smoking.

Overall, the present study demonstrates that while there are differences in the expression of many relevant oxidant-related genes in the small airway epithelium of smokers compared to nonsmokers, there are far fewer differences in gene expression in alveolar macrophages. These findings support the notion that alveolar macrophages are not "diseased" in the lungs of smokers. On the other hand, changes in small airway epithelium gene expression are a harbinger of smoking-related disease and these cells become deranged and disorganized in smoke-exposed individuals. The observation of differential responses of these two cell types to cigarette smoke exposure is consistent with this explanation.

## Competing interests

The authors declare that they have no competing interests.

## Authors' contributions

BC participated in study design, collection of biological samples, gene expression analysis and interpretation, statistical analyses, TaqMan RT PCR analyses and drafted the manuscript. BGH participated in the collection of biological samples and study design. NH participated in gene expression analysis and interpretation. TOC participated in data analysis, statistical analysis and provided helpful discussion. PC: provided useful discussion and review and helped develop the oxidation transcriptome. RGC conceived the study, oversaw collection of biological samples, participated in study design and coordination and helped with drafting the manuscript.

## Supplementary Material

Additional file 1 **Expression and Fold Change Healthy Smokers Compared to Nonsmokers of Oxidant-related Genes in Small Airway Epithelium and Alveolar Macrophages**. % expression of oxidant-related genes in small airway epithelium and alveolar macrophages from the same healthy nonsmokers and healthy smokers. Where more than one probe set identification exists for a gene, the probe set identification with the highest expression is presented here. Click here for file

Additional file 2 **Differential Expression of Oxidant-related Genes in Alveolar Macrophages and Small Airway Epithelium from the Same Healthy Nonsmokers**. Expression (as detection call of present) in alveolar macrophages (AM) and small airway epithelium (SAE) of healthy nonsmokers. Click here for file

Additional file 3 **Relative gene expression levels of oxidant-related genes in small airway epithelium and alveolar macrophages of healthy nonsmokers**. The categories of oxidant-related genes together with each individual gene in that category are presented on the ordinate and the average relative expression (log_10 _scale) on the abscissa. Click here for file

Additional file 4 **Differential Expression of Oxidant-related Genes in Alveolar Macrophages and Small Airway Epithelium from the Same Healthy Smokers**. Expression (as detection call of present) in alveolar macrophages (AM) and small airway epithelium (SAE) of healthy smokers. Click here for file

Additional file 5 **Relative gene expression levels of oxidant-related genes in small airway epithelium and alveolar macrophages of healthy smokers**. The categories of oxidant-related genes together with each individual gene in that category are presented on the ordinate and the average relative expression (log_10 _scale) on the abscissa. Click here for file
