# Supplementary material for: Disparate oxidant gene expression of airway epithelium compared to alveolar macrophages in smokers
Source: Respir Res. 2009 Nov 17;10(1):111. doi: 10.1186/1465-9921-10-111 (PMC2787510; doi:10.1186/1465-9921-10-111)
Supplement: Additional file 1 — Expression and Fold Change Healthy Smokers Compared to Nonsmokers of Oxidant-related Genes in Small Airway Epithelium and Alveolar Macrophages. % expression of oxidant-related genes in small airway epithelium and alveolar macrophages from the same healthy nonsmokers and healthy smokers. Where more than one probe set identification exists for a gene, the probe set identification with the highest expression is presented here. [file 1465-9921-10-111-S1.PDF]

**Additional File 1. Expression and Fold Change Healthy Smokers Compared to Nonsmokers of Oxidant-related Genes in Small Airway Epithelium and Alveolar Macrophages[1]**

| Category | Probe Set ID | Gene symbol | Gene title | Small airway epithelium: Nonsmokers % expression | Small airway epithelium: Healthy smokers % expression | Alveolar macrophages: Nonsmokers % expression | Alveolar macrophages: Healthy smokers % expression | Small airway epithelium: Fold-change[2] | Small airway epithelium: p value | Alveolar macrophages: Fold-change | Alveolar macrophages: p value |
|---|---|---|---|---|---|---|---|---|---|---|---|
| **Glutathione metabolism** | 202804_at | ABCC1 | ATP-binding cassette, sub-family C, member 1 | 100 | 100 | 100 | 100 | 1.22 | 0.204 | 1.02 | 0.946 |
| | 202275_at | G6PD | glucose-6-phosphate dehydrogenase | 42 | 77 | 100 | 100 | 1.78 | 0.015 | 1.42 | 0.143 |
| | 202923_s_at | GCLC | glutamate-cysteine ligase, catalytic subunit | 100 | 100 | 100 | 100 | 1.50 | 0.005 | 1.67 | 0.007 |
| | 203925_at | GCLM | glutamate-cysteine ligase, modifier subunit | 100 | 100 | 100 | 100 | 1.28 | 0.110 | 1.19 | 0.555 |
| | 207131_x_at | GGT1 | gamma-glutamyltransferase 1 | 95 | 77 | 100 | 97 | 1.01 | 0.985 | 1.30 | 0.342 |
| | 200736_s_at | GPX1 | glutathione peroxidase 1 | 100 | 100 | 100 | 100 | -1.14 | 0.414 | 1.20 | 0.302 |
| | 202831_at | GPX2 | glutathione peroxidase 2 | 100 | 100 | 0 | 0 | 5.00 | 0.001 | 1.16 | 0.808 |
| | 201348_at | GPX3 | glutathione peroxidase 3 | 100 | 100 | 100 | 100 | 1.27 | 0.333 | 1.19 | 0.625 |
| | 201106_at | GPX4 | glutathione peroxidase 4 | 100 | 100 | 100 | 100 | -1.11 | 0.529 | 1.09 | 0.684 |
| | 213170_at | GPX7 | glutathione peroxidase 7 | 95 | 73 | 63 | 67 | -1.31 | 0.074 | 1.07 | 0.853 |
| | 205770_at | GSR | glutathione reductase | 100 | 100 | 100 | 100 | 1.60 | 0.013 | 1.41 | 0.093 |
| | 211630_s_at | GSS | glutathione synthetase | 100 | 100 | 100 | 100 | -1.01 | 0.970 | 1.06 | 0.808 |
| | 215766_at | GSTA1 | Glutathione S-transferase A1 | 100 | 100 | 100 | 100 | -1.20 | 0.094 | -1.06 | 0.687 |
| | 222102_at | GSTA3 | glutathione S-transferase A3 | 100 | 100 | 0 | 0 | 1.20 | 0.428 | -1.09 | 0.875 |
| | 202967_at | GSTA4 | glutathione S-transferase A4 | 100 | 100 | 100 | 97 | 1.18 | 0.188 | -1.11 | 0.562 |
| | 217751_at | GSTK1 | glutathione S-transferase kappa 1 | 100 | 100 | 100 | 100 | -1.07 | 0.603 | 1.13 | 0.562 |
| | 215333_x_at | GSTM1 | glutathione S-transferase M1 | 100 | 100 | 95 | 97 | -1.19 | 0.333 | 1.25 | 0.548 |
| | 204418_x_at | GSTM2 | glutathione S-transferase M2 | 100 | 100 | 100 | 97 | -1.14 | 0.409 | 1.20 | 0.533 |
| | 202554_s_at | GSTM3 | glutathione S-transferase M3 | 95 | 100 | 100 | 100 | 1.10 | 0.742 | -1.18 | 0.719 |
| | 210912_x_at | GSTM4 | glutathione S-transferase M4 | 100 | 93 | 100 | 97 | -1.18 | 0.199 | 1.15 | 0.552 |
| | 201470_at | GSTO1 | glutathione S-transferase omega 1 | 100 | 100 | 100 | 100 | -1.06 | 0.768 | -1.01 | 0.937 |
| | 227163_at | GSTO2 | glutathione S-transferase omega 2 | 100 | 100 | 5 | 3 | -1.17 | 0.142 | -1.87 | 0.099 |
| | 200824_at | GSTP1 | glutathione S-transferase pi | 100 | 100 | 100 | 100 | -1.01 | 0.965 | 1.10 | 0.610 |
| | 203815_at | GSTT1 | glutathione S-transferase theta 1 | 68 | 77 | 74 | 77 | 1.35 | 0.817 | 1.63 | 0.684 |
| | 205439_at | GSTT2 | glutathione S-transferase theta 2 | 53 | 30 | 47 | 60 | -2.23 | 0.149 | -1.41 | 0.520 |
| | 209531_at | GSTZ1 | glutathione transferase zeta 1 | 100 | 100 | 95 | 100 | -1.12 | 0.489 | 1.01 | 0.994 |
| | 201193_at | IDH1 | isocitrate dehydrogenase 1, soluble | 100 | 100 | 100 | 100 | 1.01 | 0.960 | 1.39 | 0.143 |
| | 210046_s_at | IDH2 | isocitrate dehydrogenase 2 | 100 | 100 | 100 | 100 | 1.09 | 0.742 | 1.70 | 0.075 |
| | 202069_s_at | IDH3A | isocitrate dehydrogenase 3 alpha | 100 | 100 | 100 | 100 | -1.02 | 0.924 | 1.13 | 0.460 |
| | 201509_at | IDH3B | isocitrate dehydrogenase 3 beta | 100 | 100 | 100 | 100 | -1.12 | 0.409 | 1.19 | 0.335 |

**Additional File 1. Expression and Fold Change Healthy Smokers Compared to Nonsmokers of Oxidant-related Genes in Small Airway Epithelium and Alveolar Macrophages**[1] (cont. page 2)

| | | | | Small airway epithelium | | Alveolar macrophages | | Small airway epithelium | | Alveolar macrophages | |
|---|---|---|---|---|---|---|---|---|---|---|---|
| **Category** | **Probe Set ID** | **Gene symbol** | **Gene title** | **Nonsmokers % expression** | **Healthy smokers % expression** | **Nonsmokers % expression** | **Healthy smokers % expression** | **Fold-change**[2] | **p value** | **Fold-change** | **p value** |
| | 202471_s_at | IDH3G | isocitrate dehydrogenase 3 gamma | 100 | 100 | 100 | 100 | -1.20 | 0.387 | 1.15 | 0.463 |
| | 1565162_s_at | MGST1 | microsomal glutathione S-transferase 1 | 100 | 100 | 100 | 100 | 1.03 | 0.899 | 1.14 | 0.392 |
| | 204168_at | MGST2 | microsomal glutathione S-transferase 2 | 100 | 100 | 100 | 100 | -1.10 | 0.458 | 1.24 | 0.328 |
| | 201403_s_at | MGST3 | microsomal glutathione S-transferase 3 | 100 | 100 | 100 | 100 | -1.05 | 0.811 | 1.06 | 0.649 |
| | 242617_at | TMED8 | Transmembrane emp24 protein transport domain containing 8 | 100 | 97 | 100 | 100 | 1.16 | 0.419 | 1.28 | 0.214 |
| **Redox balance** | 210505_at | ADH7 | alcohol dehydrogenase 7 (class IV), mu or sigma polypeptide | 100 | 100 | 5 | 10 | 5.37 | 0.001 | -1.28 | 0.562 |
| | 201272_at | AKR1B1 | aldo-keto reductase family 1, member B1 | 100 | 100 | 100 | 100 | 1.72 | 0.001 | -1.15 | 0.328 |
| | 209160_at | AKR1C3 | aldo-keto reductase family 1, member C3 | 100 | 100 | 100 | 100 | 2.53 | 0.001 | 1.16 | 0.439 |
| **Catalase/ SOD** | 201432_at | CAT | catalase | 100 | 100 | 100 | 100 | 1.05 | 0.808 | 1.11 | 0.591 |
| | 200642_at | SOD1 | superoxide dismutase 1, soluble | 100 | 100 | 100 | 100 | 1.07 | 0.649 | 1.08 | 0.631 |
| | 216841_s_at | SOD2 | superoxide dismutase 2 | 100 | 100 | 100 | 100 | -1.19 | 0.338 | -2.06 | 0.006 |
| | 205236_x_at | SOD3 | superoxide dismutase 3 | 5 | 0 | 5 | 0 | -1.05 | 0.899 | 1.11 | 0.780 |
| **Other oxidants scavengers** | 227253_at | CP | ceruloplasmin | 100 | 100 | 63 | 47 | -1.10 | 0.648 | -1.64 | 0.294 |
| | 219933_at | GLRX2 | glutaredoxin 2 | 100 | 100 | 100 | 100 | -1.18 | 0.294 | 1.10 | 0.512 |
| | 210682_at | LPO | lactoperoxidase | 0 | 0 | 0 | 0 | 1.09 | 0.899 | -1.20 | 0.742 |
| | 202018_s_at | LTF | lactotransferrin | 100 | 0 | 87 | 0 | -3.16 | 0.000 | -1.070 | 0.772 |
| | 216336_x_at | MT1A | metallothionein 1A | 100 | 100 | 100 | 100 | -1.47 | 0.005 | -1.57 | 0.089 |
| | 212859_x_at | MT1E | metallothionein 1E | 100 | 100 | 100 | 100 | -1.75 | 0.001 | -1.54 | 0.075 |
| | 217165_x_at | MT1F | metallothionein 1F | 100 | 100 | 100 | 100 | -1.99 | 0.001 | -1.51 | 0.151 |
| | 217546_at | MT1M | metallothionein 1M | 21 | 10 | 100 | 97 | -2.49 | 0.015 | -2.91 | 0.093 |
| | 208581_x_at | MT1X | metallothionein 1X | 100 | 100 | 100 | 100 | -1.56 | 0.002 | -1.39 | 0.211 |
| | 212185_x_at | MT2A | metallothionein 2A | 100 | 100 | 100 | 100 | -1.64 | 0.001 | -1.32 | 0.213 |
| | 208680_at | PRDX1 | peroxiredoxin 1 | 100 | 100 | 100 | 100 | 1.40 | 0.038 | -1.01 | 0.965 |
| | 39729_at | PRDX2 | peroxiredoxin 2 | 100 | 100 | 100 | 100 | -1.20 | 0.180 | -1.03 | 0.963 |
| | 201619_at | PRDX3 | peroxiredoxin 3 | 100 | 100 | 100 | 100 | -1.23 | 0.094 | -1.02 | 0.924 |
| | 201923_at | PRDX4 | peroxiredoxin 4 | 100 | 100 | 100 | 100 | -1.11 | 0.409 | 1.17 | 0.282 |
| | 222994_at | PRDX5 | peroxiredoxin 5 | 100 | 100 | 100 | 100 | -1.14 | 0.377 | 1.20 | 0.460 |
| | 200845_s_at | PRDX6 | peroxiredoxin 6 | 100 | 100 | 100 | 100 | -1.04 | 0.817 | -1.06 | 0.742 |
| | 203400_s_at | TF | transferrin | 42 | 37 | 0 | 3 | -1.23 | 0.808 | 1.41 | 0.562 |
| | 207332_s_at | TFRC | transferrin receptor | 100 | 100 | 100 | 100 | 1.10 | 0.711 | 1.01 | 0.971 |
| | 210614_at | TTPA | tocopherol transfer protein | 11 | 0 | 0 | 0 | -1.05 | 0.950 | 1.28 | 0.693 |
| | 216609_at | TXN | Thioredoxin | 100 | 100 | 100 | 100 | 1.44 | 0.199 | -1.68 | 0.224 |
| | 209077_at | TXN2 | thioredoxin 2 | 100 | 100 | 100 | 100 | -1.15 | 0.409 | 1.16 | 0.263 |

**Additional File 1. Expression and Fold Change Healthy Smokers Compared to Nonsmokers of Oxidant-related Genes in Small Airway Epithelium and Alveolar Macrophages[1]** (cont. page 3)

| Category | Probe Set ID | Gene symbol | Gene title | Small airway epithelium Nonsmokers % expression | Small airway epithelium Healthy smokers % expression | Alveolar macrophages Nonsmokers % expression | Alveolar macrophages Healthy smokers % expression | Small airway epithelium Fold-change[2] | Small airway epithelium p value | Alveolar macrophages Fold-change | Alveolar macrophages p value |
|---|---|---|---|---|---|---|---|---|---|---|---|
| | 201266_at | TXNRD1 | thioredoxin reductase 1 | 100 | 100 | 100 | 100 | 1.87 | 0.001 | 1.28 | 0.163 |
| | 211177_s_at | TXNRD2 | thioredoxin reductase 2 | 37 | 47 | 79 | 97 | -1.22 | 0.338 | 1.21 | 0.509 |
| | 59631_at | TXNRD3 | thioredoxin reductase 3 | 100 | 97 | 79 | 77 | -1.11 | 0.416 | 1.09 | 0.843 |
| **Pentose phosphate cycle** | 208308_s_at | GPI | glucose phosphate isomerase | 100 | 100 | 100 | 100 | 1.01 | 0.952 | 1.14 | 0.579 |
| | 210976_s_at | PFKM | phosphofructokinase, muscle | 100 | 100 | 100 | 97 | -1.02 | 0.899 | 1.00 | 0.997 |
| | 201037_at | PFKP | phosphofructokinase, platelet | 100 | 100 | 100 | 100 | -1.13 | 0.545 | 1.09 | 0.781 |
| | 201118_at | PGD | phosphogluconate dehydrogenase | 100 | 100 | 100 | 100 | 1.66 | 0.008 | 1.17 | 0.352 |
| | 218388_at | PGLS | 6-phosphogluconolactonase | 100 | 100 | 100 | 100 | -1.01 | 0.958 | 1.25 | 0.477 |
| | 201968_s_at | PGM1 | phosphoglucomutase 1 | 100 | 100 | 100 | 100 | -1.18 | 0.280 | 1.05 | 0.815 |
| | 213093_at | PRKCA | protein kinase C, alpha | 100 | 100 | 95 | 100 | -1.52 | 0.001 | 1.86 | 0.037 |
| | 209440_at | PRPS1 | phosphoribosyl pyrophosphate synthetase 1 | 100 | 100 | 100 | 100 | -1.02 | 0.899 | 1.04 | 0.852 |
| | 230352_at | PRPS2 | Phosphoribosyl pyrophosphate synthetase 2 | 100 | 100 | 100 | 100 | -1.47 | 0.204 | -1.43 | 0.445 |
| | 225040_s_at | RPE | ribulose-5-phosphate-3-epimerase | 100 | 100 | 100 | 100 | -1.06 | 0.820 | 1.18 | 0.277 |
| | 212973_at | RPIA | ribose 5-phosphate isomerase A | 100 | 100 | 100 | 100 | -1.16 | 0.393 | -1.12 | 0.392 |
| | 206283_s_at | TAL1 | T-cell acute lymphocytic leukemia 1 | 42 | 53 | 16 | 20 | 1.26 | 0.534 | 1.16 | 0.781 |
| | 226835_s_at | TALDO1 | transaldolase 1 | 100 | 100 | 100 | 100 | -1.17 | 0.338 | 1.27 | 0.266 |
| | 208699_x_at | TKT | transketolase | 100 | 100 | 100 | 100 | 1.79 | 0.008 | 1.52 | 0.198 |
| **Xenobiotic metabolism** | 205749_at | CYP1A1 | cytochrome P450, family 1, subfamily A, polypeptide 1 | 0 | 43 | 0 | 10 | 9.07 | 0.001 | 1.29 | 0.699 |
| | 207608_x_at | CYP1A2 | cytochrome P450, family 1, subfamily A, polypeptide 2 | 68 | 70 | 47 | 53 | 1.01 | 0.970 | 1.34 | 0.460 |
| | 202437_s_at | CYP1B1 | cytochrome P450, family 1, subfamily B, polypeptide 1 | 32 | 97 | 100 | 100 | 25.41 | 0.001 | 1.69 | 0.143 |
| | 1494_f_at | CYP2A6 | cytochrome P450, family 2, subfamily A, polypeptide 6 | 100 | 100 | 32 | 50 | -1.26 | 0.103 | 1.41 | 0.143 |
| | 216340_s_at | CYP2A7P1 | cytochrome P450, family 2, subfamily A, polypeptide 7 pseudogene 1 | 0 | 3 | 0 | 3 | 1.11 | 0.820 | -1.15 | 0.762 |
| | 207718_x_at | CYP2A13 | cytochrome P450, family 2, subfamily A, polypeptide 13 | 89 | 73 | 0 | 3 | -1.39 | 0.015 | 1.48 | 0.472 |
| | 217133_x_at | CYP2B6 | cytochrome P450, family 2, subfamily B, polypeptide 6 | 95 | 90 | 47 | 50 | -1.14 | 0.587 | -1.23 | 0.277 |
| | 210272_at | CYP2B7P1 | cytochrome P450, family 2, subfamily B, polypeptide 7 pseudogene 1 | 100 | 100 | 0 | 7 | -1.10 | 0.759 | -1.41 | 0.548 |
| | 208147_s_at | CYP2C8 | cytochrome P450, family 2, subfamily C, polypeptide 8 | 100 | 100 | 0 | 0 | 1.11 | 0.647 | 1.17 | 0.825 |
| | 216025_x_at | CYP2C9 | cytochrome P450, family 2, subfamily C, polypeptide 9 | 100 | 100 | 21 | 17 | 1.18 | 0.301 | -1.11 | 0.857 |

**Additional File 1. Expression and Fold Change Healthy Smokers Compared to Nonsmokers of Oxidant-related Genes in Small Airway Epithelium and Alveolar Macrophages[1]** (cont. page 4)

| | | | | Small airway epithelium | | Alveolar macrophages | | Small airway epithelium | | Alveolar macrophages | |
|---|---|---|---|---|---|---|---|---|---|---|---|
| Category | Probe Set ID | Gene symbol | Gene title | Nonsmokers % expression | Healthy smokers % expression | Nonsmokers % expression | Healthy smokers % expression | Fold-change[2] | p value | Fold-change | p value |
| | 208126_s_at | CYP2C18 | cytochrome P450, family 2, subfamily C, polypeptide 18 | 68 | 67 | 0 | 0 | 1.08 | 0.808 | 1.82 | 0.152 |
| | 216058_s_at | CYP2C19 | cytochrome P450, family 2, subfamily C, polypeptide 19 | 5 | 10 | 0 | 0 | 1.42 | 0.428 | -1.09 | 0.875 |
| | 207498_s_at | CYP2D6 | cytochrome P450, family 2, subfamily D, polypeptide 6 | 47 | 63 | 58 | 43 | -1.04 | 0.899 | -1.18 | 0.591 |
| | 209975_at | CYP2E1 | cytochrome P450, family 2, subfamily E, polypeptide 1 | 79 | 53 | 0 | 3 | -1.54 | 0.070 | -1.54 | 0.341 |
| | 205073_at | CYP2J2 | cytochrome P450, family 2, subfamily J, polypeptide 2 | 100 | 100 | 0 | 0 | -1.25 | 0.047 | -1.40 | 0.512 |
| | 227109_at | CYP2R1 | cytochrome P450, family 2, subfamily R, polypeptide 1 | 100 | 100 | 100 | 100 | 1.01 | 0.952 | 1.06 | 0.745 |
| | 223385_at | CYP2S1 | cytochrome P450, family 2, subfamily S, polypeptide 1 | 100 | 97 | 74 | 97 | -1.01 | 0.988 | 1.83 | 0.093 |
| | 226393_at | CYP2U1 | cytochrome P450, family 2, subfamily U, polypeptide 1 | 100 | 100 | 89 | 100 | 1.01 | 0.924 | -1.27 | 0.335 |
| | 220562_at | CYP2W1 | cytochrome P450, family 2, subfamily W, polypeptide 1 | 42 | 20 | 0 | 0 | -1.41 | 0.236 | -1.08 | 0.910 |
| | 205999_x_at | CYP3A4 | cytochrome P450, family 3, subfamily A, polypeptide 4 | 11 | 3 | 53 | 17 | -1.01 | 0.988 | -1.61 | 0.214 |
| | 211440_x_at | CYP3A43 | cytochrome P450, family 3, subfamily A, polypeptide 43 | 11 | 3 | 5 | 3 | -1.11 | 0.817 | 1.16 | 0.852 |
| | 205765_at | CYP3A5 | cytochrome P450, family 3, subfamily A, polypeptide 5 | 26 | 57 | 42 | 13 | 1.08 | 0.820 | -1.98 | 0.400 |
| | 205939_at | CYP3A7 | cytochrome P450, family 3, subfamily A, polypeptide 7 | 5 | 10 | 58 | 10 | 1.13 | 0.820 | -3.69 | 0.030 |
| | 207407_x_at | CYP4A11 | cytochrome P450, family 4, subfamily A, polypeptide 11 | 0 | 23 | 5 | 17 | 1.35 | 0.419 | -1.03 | 0.965 |
| | 217319_x_at | CYP4A22 | cytochrome P450, family 4, subfamily A, polypeptide 22 | 0 | 3 | 5 | 27 | 1.70 | 0.182 | 1.12 | 0.802 |
| | 210096_at | CYP4B1 | cytochrome P450, family 4, subfamily B, polypeptide 1 | 100 | 100 | 0 | 3 | -1.39 | 0.005 | 2.03 | 0.147 |
| | 210452_x_at | CYP4F2 | cytochrome P450, family 4, subfamily F, polypeptide 2 | 0 | 0 | 0 | 0 | 1.92 | 0.094 | -1.25 | 0.405 |
| | 206515_at | CYP4F3 | cytochrome P450, family 4, subfamily F, polypeptide 3 | 95 | 93 | 5 | 7 | 2.27 | 0.001 | -1.06 | 0.946 |
| | 210576_at | CYP4F8 | cytochrome P450, family 4, subfamily F, polypeptide 8 | 0 | 0 | 0 | 0 | 1.09 | 0.886 | -1.64 | 0.266 |

**Additional File 1. Expression and Fold Change Healthy Smokers Compared to Nonsmokers of Oxidant-related Genes in Small Airway Epithelium and Alveolar Macrophages**[1] (cont. page 5)

| | | | | Small airway epithelium | | Alveolar macrophages | | Small airway epithelium | | Alveolar macrophages | |
|---|---|---|---|---|---|---|---|---|---|---|---|
| **Category** | **Probe Set ID** | **Gene symbol** | **Gene title** | **Nonsmokers % expression** | **Healthy smokers % expression** | **Nonsmokers % expression** | **Healthy smokers % expression** | **Fold-change**[2] | **p value** | **Fold-change** | **p value** |
| | 206153_at | CYP4F11 | cytochrome P450, family 4, subfamily F, polypeptide 11 | 79 | 90 | 11 | 7 | 3.21 | 0.013 | -1.77 | 0.472 |
| | 206539_s_at | CYP4F12 | cytochrome P450, family 4, subfamily F, polypeptide 12 | 63 | 63 | 0 | 0 | 1.14 | 0.565 | -1.10 | 0.843 |
| | 228391_at | CYP4V2 | cytochrome P450, family 4, subfamily V, polypeptide 2 | 100 | 100 | 100 | 100 | -1.15 | 0.411 | 1.06 | 0.907 |
| | 227702_at | CYP4X1 | cytochrome P450, family 4, subfamily X, polypeptide 1 | 100 | 100 | 0 | 3 | -2.02 | 0.001 | -1.27 | 0.687 |
| | 237395_at | CYP4Z1 | cytochrome P450, family 4, subfamily Z, polypeptide 1 | 95 | 90 | 0 | 0 | -1.45 | 0.014 | -1.24 | 0.762 |
| | 1553434_at | CYP4Z2P | cytochrome P450 4Z2 pseudogene | 11 | 0 | 0 | 3 | 2.01 | 0.143 | -1.29 | 0.642 |
| | 207406_at | CYP7A1 | cytochrome P450, family 7, subfamily A, polypeptide 1 | 0 | 0 | 0 | 3 | -1.48 | 0.428 | -1.83 | 0.266 |
| | 207386_at | CYP7B1 | cytochrome P450, family 7, subfamily B, polypeptide 1 | 74 | 60 | 5 | 7 | 1.13 | 0.782 | -1.15 | 0.852 |
| | 232494_at | CYP8B1 | cytochrome P450, family 8, subfamily B, polypeptide 1 | 0 | 0 | 0 | 0 | 1.04 | 0.928 | -1.43 | 0.163 |
| | 204309_at | CYP11A1 | cytochrome P450, family 11, subfamily A, polypeptide 1 | 0 | 0 | 0 | 0 | 1.04 | 0.957 | 1.01 | 0.996 |
| | 214610_at | CYP11B1 | cytochrome P450, family 11, subfamily B, polypeptide 1 | 0 | 0 | 0 | 0 | 1.72 | 0.316 | -1.21 | 0.766 |
| | 214630_at | CYP11B2 | cytochrome P450, family 11, subfamily B, polypeptide 2 | 0 | 0 | 5 | 3 | 1.18 | 0.565 | 1.22 | 0.591 |
| | 1562573_at | CYP17A1 | cytochrome P450, family 17, subfamily A, polypeptide 1 | 0 | 7 | 5 | 0 | 1.03 | 0.970 | 1.18 | 0.762 |
| | 203475_at | CYP19A1 | cytochrome P450, family 19, subfamily A, polypeptide 1 | 5 | 3 | 5 | 0 | 1.18 | 0.811 | -1.30 | 0.687 |
| | 219565_at | CYP20A1 | cytochrome P450, family 20, subfamily A, polypeptide 1 | 95 | 100 | 100 | 100 | 1.06 | 0.808 | 1.15 | 0.406 |
| | 214622_at | CYP21A2 | cytochrome P450, family 21, subfamily A, polypeptide 2 | 0 | 0 | 0 | 0 | 1.25 | 0.533 | -1.22 | 0.508 |
| | 206504_at | CYP24A1 | cytochrome P450, family 24, subfamily A, polypeptide 1 | 79 | 80 | 0 | 3 | 1.22 | 0.680 | 1.06 | 0.946 |
| | 206424_at | CYP26A1 | cytochrome P450, family 26, subfamily A, polypeptide 1 | 37 | 63 | 0 | 0 | 2.05 | 0.045 | 1.05 | 0.946 |
| | 234721_s_at | CYP26B1 | cytochrome P450, family 26, subfamily B, polypeptide 1 | 0 | 0 | 0 | 0 | 1.24 | 0.594 | -1.23 | 0.562 |

**Additional File 1. Expression and Fold Change Healthy Smokers Compared to Nonsmokers of Oxidant-related Genes in Small Airway Epithelium and Alveolar Macrophages[1]** (cont. page 6)

| | | | | Small airway epithelium | | Alveolar macrophages | | Small airway epithelium | | Alveolar macrophages | |
|---|---|---|---|---|---|---|---|---|---|---|---|
| **Category** | **Probe Set ID** | **Gene symbol** | **Gene title** | **Nonsmokers % expression** | **Healthy smokers % expression** | **Nonsmokers % expression** | **Healthy smokers % expression** | **Fold-change[2]** | **p value** | **Fold-change** | **p value** |
| | 203979_at | CYP27A1 | cytochrome P450, family 27, subfamily A, polypeptide 1 | 68 | 63 | 100 | 100 | -1.33 | 0.218 | 1.24 | 0.152 |
| | 205676_at | CYP27B1 | cytochrome P450, family 27, subfamily B, polypeptide 1 | 0 | 3 | 63 | 70 | 1.34 | 0.470 | -1.92 | 0.143 |
| | 1553977_a_at | CYP39A1 | cytochrome P450, family 39, subfamily A, polypeptide 1 | 100 | 97 | 5 | 10 | 1.16 | 0.294 | 1.14 | 0.765 |
| | 220331_at | CYP46A1 | cytochrome P450, family 46, subfamily A, polypeptide 1 | 26 | 23 | 0 | 0 | -1.19 | 0.561 | 1.23 | 0.632 |
| | 216607_s_at | CYP51A1 | cytochrome P450, family 51, subfamily A, polypeptide 1 | 100 | 100 | 100 | 100 | -1.05 | 0.894 | 1.36 | 0.228 |
| | 202017_at | EPHX1 | epoxide hydrolase 1, microsomal (xenobiotic) | 100 | 100 | 100 | 100 | -1.08 | 0.820 | 1.39 | 0.335 |
| | 209368_at | EPHX2 | epoxide hydrolase 2, cytoplasmic | 95 | 97 | 0 | 0 | -1.19 | 0.409 | -1.46 | 0.301 |
| | 214627_at | EPX | eosinophil peroxidase | 0 | 0 | 0 | 3 | 1.09 | 0.834 | -1.43 | 0.294 |
| | 228678_at | FAM116B | family with sequence similarity 116, member B | 95 | 90 | 0 | 0 | -1.16 | 0.587 | 1.78 | 0.151 |
| | 1558549_s_at | VNN1 | vanin 1 | 53 | 43 | 74 | 97 | -1.52 | 0.218 | 2.04 | 0.081 |
| **Selenium-related** | 224888_at | SELI | selenoprotein I | 100 | 100 | 100 | 100 | 1.03 | 0.820 | 1.07 | 0.631 |
| | 223070_at | SELK | selenoprotein K | 100 | 100 | 100 | 100 | -1.13 | 0.424 | -1.16 | 0.266 |
| | 226051_at | SELM | selenoprotein M | 100 | 93 | 84 | 100 | -1.04 | 0.894 | -1.04 | 0.946 |
| | 223209_s_at | SELS | selenoprotein S | 100 | 100 | 100 | 100 | 1.03 | 0.886 | 1.02 | 0.946 |
| | 225561_at | SELT | selenoprotein T | 100 | 100 | 100 | 100 | -1.21 | 0.414 | -1.25 | 0.266 |
| | 237475_x_at | SEPP1 | Selenoprotein P, plasma, 1 | 100 | 100 | 100 | 100 | 1.10 | 0.693 | -1.05 | 0.684 |
| | 201194_at | SEPW1 | selenoprotein W, 1 | 100 | 100 | 100 | 100 | -1.17 | 0.294 | 1.23 | 0.143 |
| **Bilirubin-related** | 203771_s_at | BLVRA | biliverdin reductase A | 100 | 100 | 100 | 100 | -1.05 | 0.820 | 1.13 | 0.508 |
| | 203665_at | HMOX1 | heme oxygenase (decycling) 1 | 89 | 70 | 100 | 100 | -1.02 | 0.952 | 1.51 | 0.077 |
| | 218120_s_at | HMOX2 | heme oxygenase (decycling) 2 | 100 | 100 | 100 | 100 | -1.08 | 0.742 | 1.16 | 0.507 |
| | 209236_at | SLC23A2 | solute carrier family 23, member 2 | 95 | 90 | 89 | 100 | 1.08 | 0.680 | 1.50 | 0.038 |
| **Ascorbic acid-related** | 237799_at | SLC22A12 | solute carrier family 22, member 12 | 0 | 0 | 0 | 3 | 1.28 | 0.603 | 1.07 | 0.921 |
| | 223732_at | SLC23A1 | solute carrier family 23, member 1 | 100 | 97 | 0 | 0 | -1.38 | 0.142 | -1.32 | 0.579 |
| **Prod of free radicals** | 203949_at | MPO | myeloperoxidase | 0 | 7 | 0 | 13 | 1.26 | 0.647 | 1.24 | 0.610 |
| | 207309_at | NOS1 | nitric oxide synthase 1 (neuronal) | 0 | 0 | 11 | 23 | -1.26 | 0.470 | 1.08 | 0.852 |
| | 210037_s_at | NOS2A | nitric oxide synthase 2A (inducible, hepatocytes) | 32 | 17 | 0 | 0 | -1.89 | 0.110 | -1.18 | 0.765 |
| | 205581_s_at | NOS3 | nitric oxide synthase 3 (endothelial cell) | 0 | 10 | 0 | 0 | 1.17 | 0.820 | 1.32 | 0.520 |

[1] % expression of oxidant-related genes in small airway epithelium and alveolar macrophages from the same healthy nonsmokers (n=19) and healthy smokers (n=30). Where more than one probe set identification exists for a gene, the probe set identification with the highest expression is presented here.

[2] Fold-change represents the average expression in heathy smokers compared to the average expression in healthy nonsmokers.
