# Supplementary material for: Disparate oxidant gene expression of airway epithelium compared to alveolar macrophages in smokers
Source: Respir Res. 2009 Nov 17;10(1):111. doi: 10.1186/1465-9921-10-111 (PMC2787510; doi:10.1186/1465-9921-10-111)
Supplement: Additional file 2 — Differential Expression of Oxidant-related Genes in Alveolar Macrophages and Small Airway Epithelium from the Same Healthy Nonsmokers. Expression (as detection call of present) in alveolar macrophages (AM) and small airway epithelium (SAE) of healthy nonsmokers. [file 1465-9921-10-111-S2.PDF]

Additional File 2. Differential Expression of Oxidant-related Genes in Alveolar Macrophages and Small Airway Epithelium from the Same Healthy Nonsmokers[1]

Category	Probe set ID	Gene symbol	Gene	Alveolar macrophages % present	Airway epithelium% present	Fold-change AM/ SAE	p value
**Glutathione metabolism**	202804_at	ABCC1	ATP-binding cassette, sub-family C, member 1	100	100	4.94	<0.001
	202275_at	G6PD	glucose-6-phosphate dehydrogenase	77	100	26.21	<0.001
	202922_at	GCLC	glutamate-cysteine ligase, catalytic subunit	100	100	-2.10	<0.001
	207131_x_at	GGT1	gamma-glutamyltransferase 1	95	100	3.96	<0.001
	200736_s_at	GPX1	glutathione peroxidase 1	100	100	5.01	<0.001
	202831_at	GPX2	glutathione peroxidase 2	100	0	-9.88	<0.001
	201348_at	GPX3	glutathione peroxidase 3	100	100	4.67	<0.001
	201106_at	GPX4	glutathione peroxidase 4	100	100	2.92	<0.001
	205770_at	GSR	glutathione reductase	100	100	5.43	<0.001
	211630_s_at	GSS	glutathione synthetase	100	100	3.06	<0.001
	222102_at	GSTA3	glutathione S-transferase A3	100	0	-76.07	<0.001
	202967_at	GSTA4	glutathione S-transferase A4	100	100	-3.21	<0.001
	217751_at	GSTK1	glutathione S-transferase kappa 1	100	100	2.06	<0.001
	202554_s_at	GSTM3	glutathione S-transferase M3 (brain)	95	100	6.08	<0.001
	210912_x_at	GSTM4	glutathione S-transferase M4	100	100	2.20	<0.001
	201470_at	GSTO1	glutathione S-transferase omega 1	100	100	12.73	<0.001
	227163_at	GSTO2	glutathione S-transferase omega 2	100	5	-11.40	<0.001
	200824_at	GSTP1	glutathione S-transferase pi	100	100	-1.59	0.001
	201193_at	IDH1	isocitrate dehydrogenase 1 (NADP+),	100	100	2.63	<0.001
	202069_s_at	IDH3A	isocitrate dehydrogenase 3 (NAD+) alpha	100	100	1.89	<0.001
	201509_at	IDH3B	isocitrate dehydrogenase 3 (NAD+) beta	100	100	2.39	<0.001
	202471_s_at	IDH3G	isocitrate dehydrogenase 3 (NAD+) gamma	100	100	2.84	<0.001
	1565162_s_at	MGST1	microsomal glutathione S-transferase 1	100	100	4.40	<0.001
	201403_s_at	MGST3	microsomal glutathione S-transferase 3	100	100	4.99	<0.001
	242617_at	TMED8	Transmembrane emp24 protein transport domain containing 8	100	100	2.45	<0.001
**Redox balance**	210505_at	ADH7	alcohol dehydrogenase 7, mu or sigma polypeptide	100	5	-12.02	<0.001
	201272_at	AKR1B1	aldo-keto reductase family 1, member B1	100	100	39.42	<0.001
	209160_at	AKR1C3	aldo-keto reductase family 1, member C3	100	100	4.41	<0.001

Additional File 2. Differential Expression of Oxidant-related Genes in Alveolar Macrophages and Small Airway Epithelium from the Same Healthy Nonsmokers (cont., 2)

Category	Probe set ID	Gene symbol	Gene	Alveolar macrophages % present	Airway epithelium% present	Fold-change AM/ SAE	p value
**Catalase/ SOD**	211922_s_at	CAT	catalase	100	100	2.22	<0.001
	16841_s_at	SOD2	superoxide dismutase 2	100	100	48.67	<0.001
**Other oxidants scavengers**	227253_at	CP	ceruloplasmin (ferroxidase)	100	63	-22.66	<0.001
	219933_at	GLRX2	glutaredoxin 2	100	100	12.99	<0.001
	202018_s_at	LTF	lactotransferrin	100	0	-271.28	<0.001
	216336_x_at	MT1A	metallothionein 1A	100	100	11.38	<0.001
	212859_x_at	MT1E	metallothionein 1E	100	100	6.64	<0.001
	213629_x_at	MT1F	metallothionein 1F	100	100	21.70	<0.001
	217546_at	MT1M	metallothionein 1M	21	100	202.48	<0.001
	204326_x_at	MT1X	metallothionein 1X	100	100	10.15	<0.001
	212185_x_at	MT2A	metallothionein 2A	100	100	12.09	<0.001
	208680_at	PRDX1	peroxiredoxin 1	100	100	3.35	<0.001
	39729_at	PRDX2	peroxiredoxin 2	100	100	-4.10	<0.001
	201619_at	PRDX3	peroxiredoxin 3	100	100	1.85	<0.001
	201923_at	PRDX4	peroxiredoxin 4	100	100	1.84	<0.001
	1560587_s_at	PRDX5	peroxiredoxin 5	100	100	-3.23	<0.001
	200844_s_at	PRDX6	peroxiredoxin 6	100	100	1.50	0.001
	208691_at	TFRC	transferrin receptor	100	100	14.48	<0.001
	208864_s_at	TXN	thioredoxin	100	100	4.59	<0.001
	209077_at	TXN2	thioredoxin 2	100	100	1.62	0.004
	201266_at	TXNRD1	thioredoxin reductase 1	100	100	5.66	<0.001
	211177_s_at	TXNRD2	thioredoxin reductase 2	37	79	3.51	0.002
	59631_at	TXNRD3	thioredoxin reductase 3	100	79	-1.72	<0.001
	1558549_s_at	VNN1	vanin 1	53	74	3.56	0.002
**Pentose phosphate cycle**	208308_s_at	GPI	glucose phosphate isomerase	100	100	6.19	<0.001
	210976_s_at	PFKM	phosphofructokinase, muscle	100	100	-2.88	<0.001
	201037_at	PFKP	phosphofructokinase, platelet	100	100	2.66	<0.001
	201118_at	PGD	phosphogluconate dehydrogenase	100	100	17.10	<0.001
	218388_at	PGLS	6-phosphogluconolactonase	100	100	3.07	<0.001
	201968_s_at	PGM1	phosphoglucomutase 1	100	100	2.37	<0.001
	213093_at	PRKCA	protein kinase C, alpha	100	95	-2.11	<0.001
	230352_at	PRPS2	Phosphoribosyl pyrophosphate synthetase 2	100	100	3.06	0.037

Additional File 2. Differential Expression of Oxidant-related Genes in Alveolar Macrophages and Small Airway Epithelium from the Same Healthy Nonsmokers (cont., 3)

Category	Probe set ID	Gene symbol	Gene	Alveolar macrophages % present	Airway epithelium% present	Fold-change AM/ SAE	p value
	225039_at	RPE	ribulose-5-phosphate-3-epimerase	100	100	4.34	<0.001
	212973_at	RPIA	ribose 5-phosphate isomerase A	100	100	3.71	<0.001
	201463_s_at	TALDO1	transaldolase 1	100	100	6.15	<0.001
	208700_s_at	TKT	transketolase	100	100	7.27	<0.001
**Xenobiotic metabolism**	202437_s_at	CYP1B1	cytochrome P450, family 1, subfamily B, polypeptide 1	32	100	369.32	<0.001
	1494_f_at	CYP2A6	cytochrome P450, family 2, subfamily A, polypeptide 6	100	32	-2.73	<0.001
	207718_x_at	CYP2A13	cytochrome P450, family 2, subfamily A, polypeptide13	89	0	-3.21	<0.001
	217133_x_at	CYP2B6	cytochrome P450, family 2, subfamily B, polypeptide 6	95	47	-2.18	<0.001
	210272_at	CYP2B7P1	cytochrome P450, family 2, subfamily B, polypeptide 7 pseudogene 1	100	0	-21.66	<0.001
	208126_s_at	CYP2C18	cytochrome P450, family 2, subfamily C, polypeptide 18	68	0	-11.17	<0.001
	208147_s_at	CYP2C8	cytochrome P450, family 2, subfamily C, polypeptide 8	100	0	-9.75	<0.001
	216025_x_at	CYP2C9	cytochrome P450, family 2, subfamily C, polypeptide 9	100	21	-1.82	<0.001
	209975_at	CYP2E1	cytochrome P450, family 2, subfamily E, polypeptide 1	79	0	-2.47	<0.001
	205073_at	CYP2J2	cytochrome P450, family 2, subfamily J, polypeptide 2	100	0	-38.51	<0.001
	227109_at	CYP2R1	cytochrome P450, family 2, subfamily R, polypeptide 1	100	100	2.91	<0.001
	223385_at	CYP2S1	cytochrome P450, family 2, subfamily S, polypeptide 1	100	74	-2.11	<0.001
	205999_x_at	CYP3A4	cytochrome P450, family 3, subfamily A, polypeptide 4	11	53	5.47	<0.001
	205939_at	CYP3A7	cytochrome P450, family 3, subfamily A, polypeptide 7	5	58	10.61	0.001
	1555497_a_at	CYP4B1	cytochrome P450, family 4, subfamily B, polypeptide 1	100	0	-92.38	<0.001
	206539_s_at	CYP4F12	cytochrome P450, family 4, subfamily F, polypeptide 12	63	0	-7.00	<0.001
	206515_at	CYP4F3	cytochrome P450, family 4, subfamily F, polypeptide 3	95	5	-3.18	<0.001

Additional File 2. Differential Expression of Oxidant-related Genes in Alveolar Macrophages and Small Airway Epithelium from the Same Healthy Nonsmokers (cont., 4)

Category	Probe set ID	Gene symbol	Gene	Alveolar macrophages % present	Airway epithelium% present	Fold-change AM/ SAE	p value
	226745_at	CYP4V2	cytochrome P450, family 4, subfamily V, polypeptide 2	100	100	1.55	0.001
	227702_at	CYP4X1	cytochrome P450, family 4, subfamily X, polypeptide 1	100	0	-279.05	<0.001
	237395_at	CYP4Z1	cytochrome P450, family 4, subfamily Z, polypeptide 1	95	0	-8.46	<0.001
	219565_at	CYP20A1	cytochrome P450, family 20, subfamily A, polypeptide 1	95	100	3.59	<0.001
	206504_at	CYP24A1	cytochrome P450, family 24, subfamily A, polypeptide 1	79	0	-3.82	0.002
	203979_at	CYP27A1	cytochrome P450, family 27, subfamily A, polypeptide 1	68	100	39.58	<0.001
	205676_at	CYP27B1	cytochrome P450, family 27, subfamily B, polypeptide 1	0	63	28.19	<0.001
	1553977_a_at	CYP39A1	cytochrome P450, family 39, subfamily A, polypeptide 1	100	5	-4.66	<0.001
	202314_at	CYP51A1	cytochrome P450, family 51, subfamily A, polypeptide 1	100	100	-1.93	<0.001
	209368_at	EPHX2	epoxide hydrolase 2, cytoplasmic	95	0	-32.83	<0.001
	228678_at	FAM116B	family with sequence similarity 116, member B	95	0	-11.77	<0.001
**Selenium-related**	1555274_a_at	SELI	selenoprotein I	100	100	3.62	<0.001
	223070_at	SELK	selenoprotein K	100	100	3.66	<0.001
	223209_s_at	SELS	selenoprotein S	100	100	2.79	<0.001
	225561_at	SELT	selenoprotein T	100	100	2.96	<0.001
	237475_x_at	SEPP1	Selenoprotein P, plasma, 1	100	100	-2.14	<0.001
	201194_at	SEPW1	selenoprotein W, 1	100	100	-2.64	<0.001
**Bilirubin-related**	203771_s_at	BLVRA	biliverdin reductase A	100	100	7.52	<0.001
	203665_at	HMOX1	heme oxygenase (decycling) 1	89	100	45.95	<0.001
	218120_s_at	HMOX2	heme oxygenase (decycling) 2	100	100	4.76	<0.001
	209236_at	SLC23A2	solute carrier family 23, member 2	95	89	2.30	<0.001
**Ascorbic acid-related**	223732_at	SLC23A1	solute carrier family 23 , member 1	100	0	-22.93	<0.001

[1] Expression (as detection call of present) in alveolar macrophages (AM) and small airway epithelium (SAE) of healthy nonsmokers (n=19).
