# Supplementary figures and images for: Disparate oxidant gene expression of airway epithelium compared to alveolar macrophages in smokers
Source: Respir Res. 2009 Nov 17;10(1):111. doi: 10.1186/1465-9921-10-111 (PMC2787510; doi:10.1186/1465-9921-10-111)

Healthy smokers

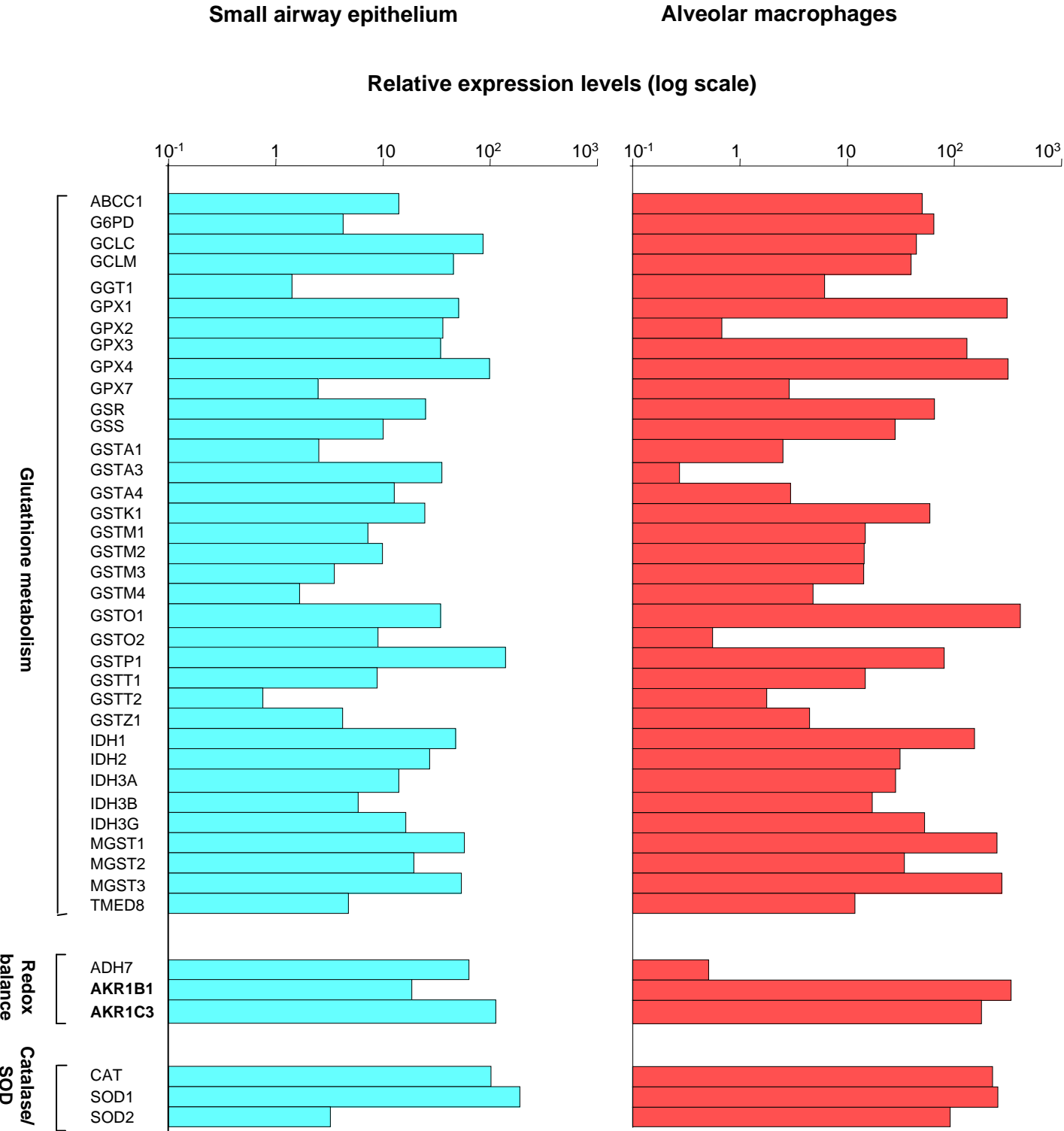

Healthy smokers

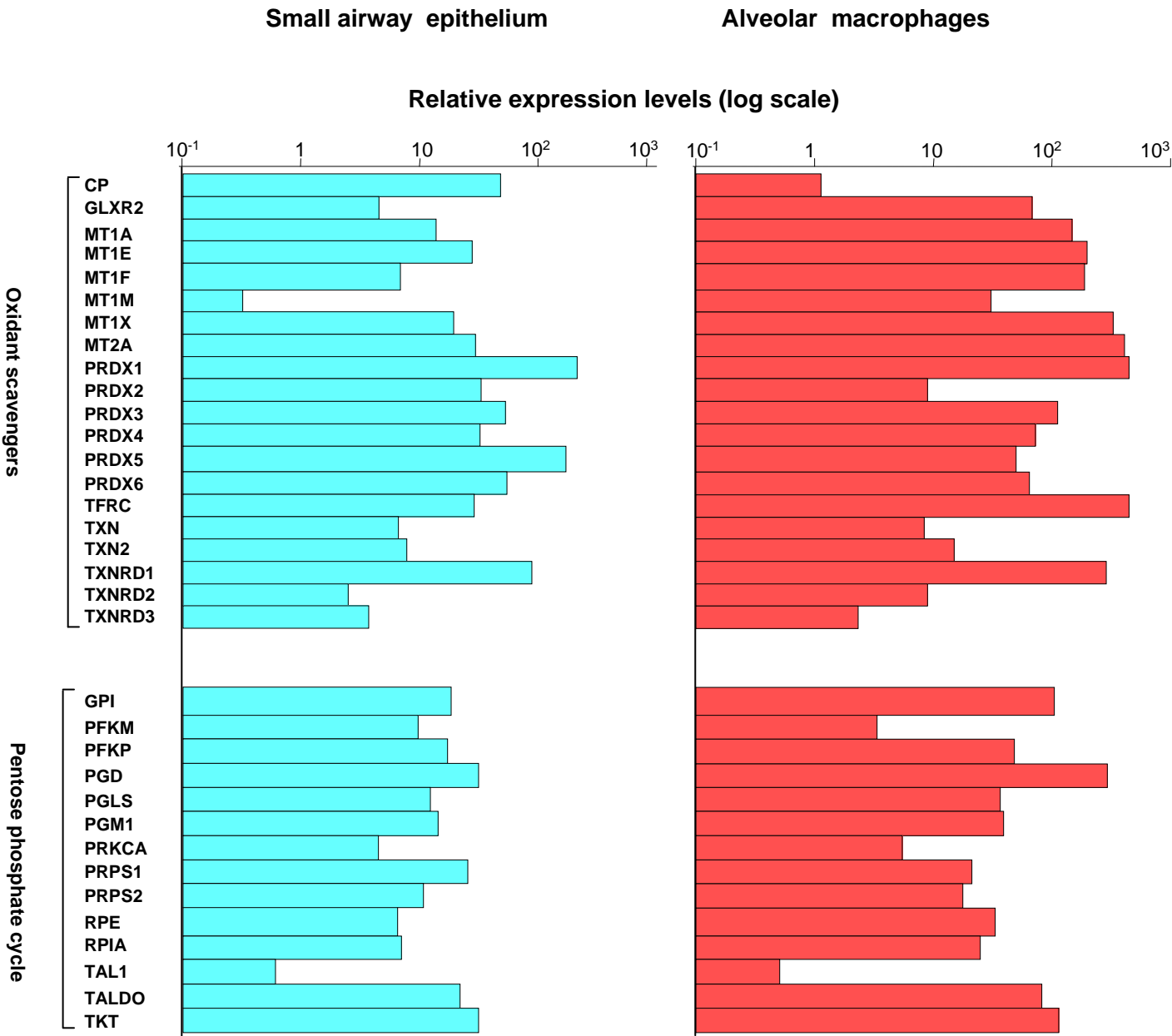

Healthy smokers

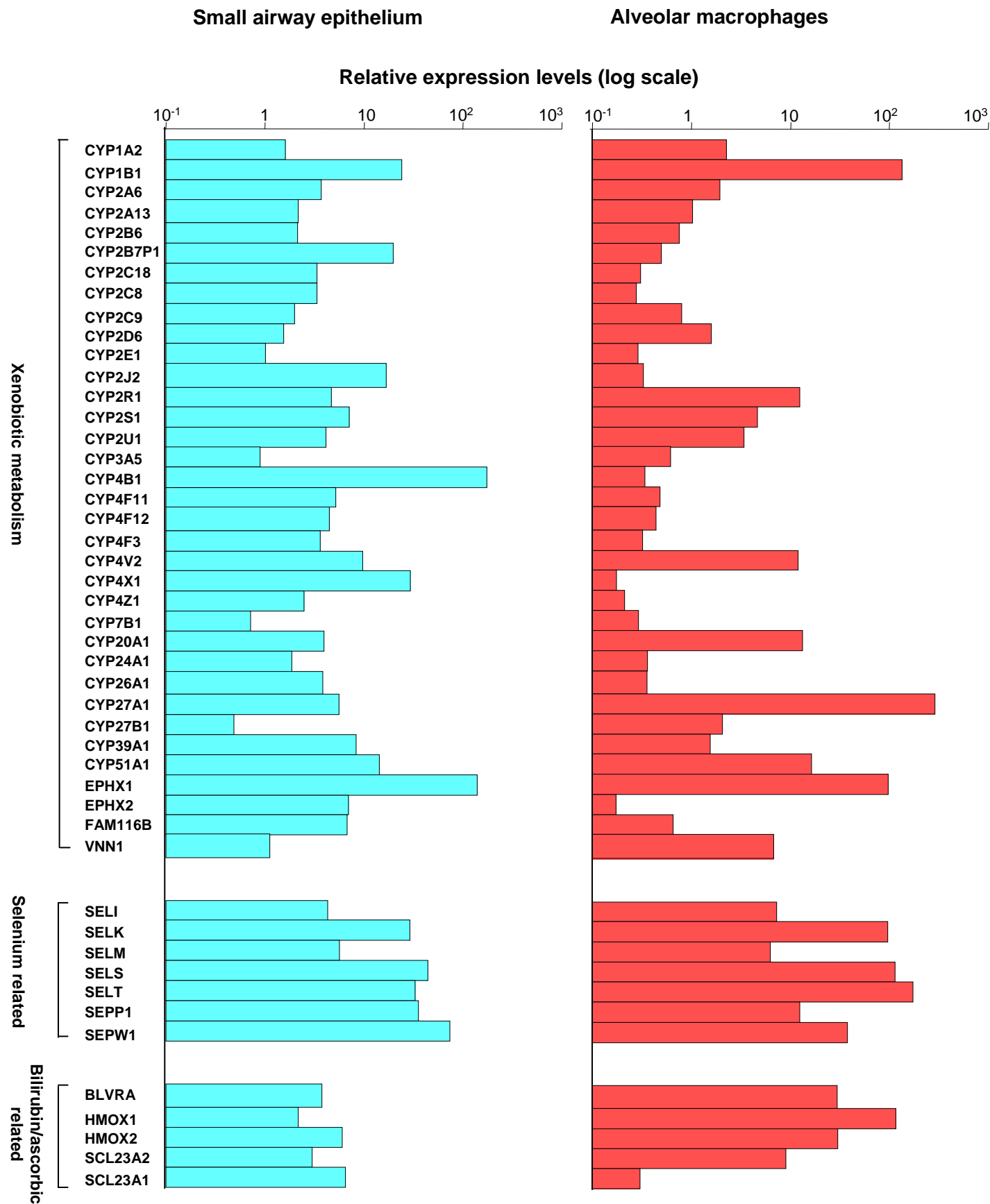

Supplement: Additional file 5 — Relative gene expression levels of oxidant-related genes in small airway epithelium and alveolar macrophages of healthy smokers. The categories of oxidant-related genes together with each individual gene in that category are presented on the ordinate and the average relative expression (log10 scale) on the abscissa. [file 1465-9921-10-111-S5.PDF]
